# The ER membrane protein complex is a transmembrane domain insertase

**DOI:** 10.1126/science.aao3099

**Published:** 2017-12-14

**Authors:** Alina Guna, Norbert Volkmar, John C. Christianson, Ramanujan S. Hegde

**Affiliations:** 1 Medical Research Council (MRC) Laboratory of Molecular Biology, Francis Crick Avenue, Cambridge Biomedical Campus, Cambridge CB2 0QH, UK; 2 Ludwig Institute for Cancer Research, University of Oxford, Old Road Campus Research Building, Headington, Oxford OX3 7DQ, UK

## Abstract

Insertion of proteins into membranes is an essential cellular process. The extensive biophysical and topological diversity of membrane proteins necessitates multiple insertion pathways that remain incompletely defined. Here we found that known membrane insertion pathways fail to effectively engage tail-anchored membrane proteins with moderately hydrophobic transmembrane domains. These proteins are instead shielded in the cytosol by calmodulin. Dynamic release from calmodulin allowed sampling of the endoplasmic reticulum (ER), where the conserved ER membrane protein complex (EMC) was shown to be essential for efficient insertion in vitro and in cells. Purified EMC in synthetic liposomes catalyzed the insertion of its substrates in a reconstituted system. Thus, EMC is a transmembrane domain insertase, a function that may explain its widely pleiotropic membrane-associated phenotypes across organisms.

The mammalian genome encodes hundreds of tail-anchored (TA) membrane proteins with essential roles in diverse processes such as vesicular trafficking, apoptosis, signal transduction, and lipid biosynthesis (*1*). A single transmembrane domain (TMD) close to the C terminus mediates posttranslational TA protein targeting and membrane insertion. Many TA proteins destined for the endoplasmic reticulum (ER) utilize the conserved TMD recognition complex (TRC) targeting pathway whose central component is TRC40 (*2*). Structural studies of Get3, the yeast homolog of TRC40, have revealed a deep hydrophobic groove that binds and shields the hydrophobic TMD of TA proteins (*3*) until their release at an ER-resident receptor complex (*4*, *5*). The surface properties of the substrate-binding groove in Get3 is consistent with biochemical studies showing a preference for TMDs of high hydrophobicity (*6*, *7*). Yet, the TMDs of ER-targeted TA proteins display a wide range of hydrophobicity and length (*1*). Whether or how the TRC pathway might handle this diversity is unclear.

The TMDs fromeight ER-destined TA proteins of widely varying biophysical properties (fig. S1) were cloned into a standardized TA protein cassette (Fig. 1A) and shown to insert into ER-derived microsomes in vitro (Fig. 1B and fig. S2).However, only the three most hydrophobic TMDs interacted efficientlywith TRC40 by native coimmunoprecipitation (Fig. 1B). Competitive inhibitors of the TRC pathway reduced insertion of only the TA proteins that efficiently engaged TRC40 (Fig. 1Candfig.S3, A toC). TheotherTAproteins were completely resistant to inhibition. One of these resistant TMDs, from the ER-resident enzyme squalene synthase (SQS), became sensitive to TRC pathway inhibition when the hydrophobicity of its TMD was increased (Fig. 1D and fig. S1). This switch from resistance to sensitivity correlated with TRC40 interaction (Fig. 1E). Even when SQS was assembled with TRC40 in a purified system, the complex dissociatedbefore appreciable insertion into ER microsomes occurred (fig. S4).

**Fig. 1:**
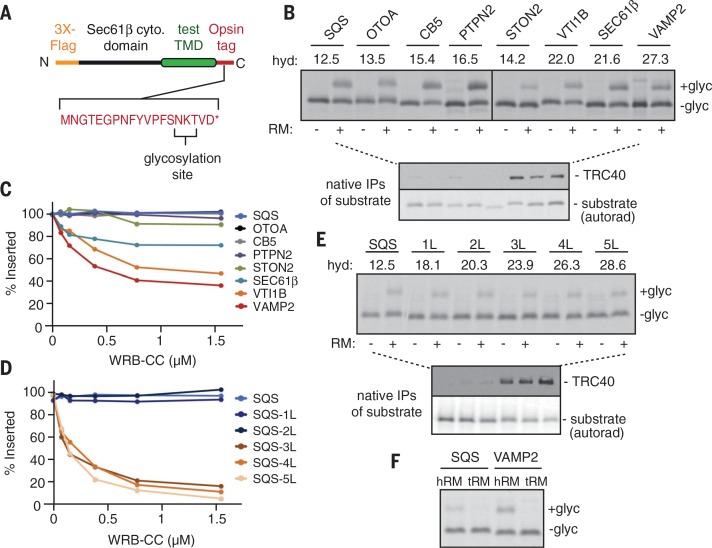
**Detection of a non-TRC insertion pathway for TA proteins**. (**A**) Diagramof the TA protein reporter cassette used for most of the analyses in this study.The asterisk at the end of the amino acid sequence indicates the stop codon. (**B**) ^35^S-methionine–labeled TA protein reporters with the indicated TMDs (see fig. S1) were translated in nucleased reticulocyte lysate (RRL) and incubated with or without canine pancreas–derived rough microsomes (RMs). Glycosylation (+ glyc) indicates successful insertion (see fig. S2). Relative hydrophobicity (hyd) values for each TMD are shown. In a parallel experiment, reactions lacking microsomes for each protein were immunoprecipitated (IP) by means of the substrate’s FLAG tag and analyzed for TRC40 association (by immunoblot) and substrate (by autoradiography, autorad). Identical results were obtained in native RRL. (**C** and **D**) Relative normalized insertion efficiencies for the indicated TA proteins with increasing amounts of the coiled-coil domain of the protein WRB (WRB-CC), a fragment of the TRC40 receptor at the ER (see fig. S3A). (**E**) An experiment as in (**B**) for a set of SQS mutants that successively increase TMD hydrophobicity through leucine (L) residue substitutions (fig. S1). (F) Analysis of SQS and VAMP2 insertion using ER microsomes from HEK293 cells (hRM) or trypsin-digested hRM (tRM; see fig. S3D). Single-letter abbreviations for the amino acid residues are as follows: D, Asp; E, Glu; F, Phe; G, Gly; K, Lys; M, Met; N, Asn; P, Pro; S, Ser; T, Thr; V, Val; and Y, Tyr.

These observations indicated that the TRC pathway only handles relatively hydrophobic ER-destined TA proteins. Based on the approximate threshold for TRC40 dependence, we estimate that around half of TA proteins are inserted into the ER via a non-TRC pathway. This conclusion is consistent with variable degrees of insertion defects seen when the TRC pathway is impaired (*8*). The mechanism of non- TRC pathway insertion remains unclear, although earlier proposals include unassisted insertion and insertion mediated by the Sec61 translocation channel (9, 10). In support of a protein-mediated process, SQS insertion into ER microsomes pretreated with trypsin was impaired (Fig. 1F and fig. S3D).We thus used SQS as amodel non-TRC substrate to identify cytosolic factor(s) thatmaintain its insertion competence and ER factor(s) needed for its insertion.

Size fractionation and chemical cross-linking were used to compare the cytosolic interactions made by the TMDs of SQS and VAMP2 (vesicleassociated membrane protein 2), an established TRC pathway substrate. As documented previously (*11*, *12*), VAMP2 interacted with each of the factors of the TRC targeting pathway: the chaperone SGTA, the Bag6 quality control complex, and TRC40 (Fig. 2A and fig. S5). The heterogeneous native size of VAMP2, as determined by sucrose gradient fractionation, reflects these multiple interactions (Fig. 2A). By contrast, SQS migrated as a smaller complex and failed to cross-link efficiently to any TRC pathway component (Fig. 2A and fig. S5). The primary cross-link seen with SQSwas a ~20 kDa Ca2+-dependent protein (Fig. 2B and fig. S5) that was identified by mass spectrometry as calmodulin (CaM), a factor shown previously to recognize hydrophobic domains in the cytosol (*13*).

**Fig. 2:**
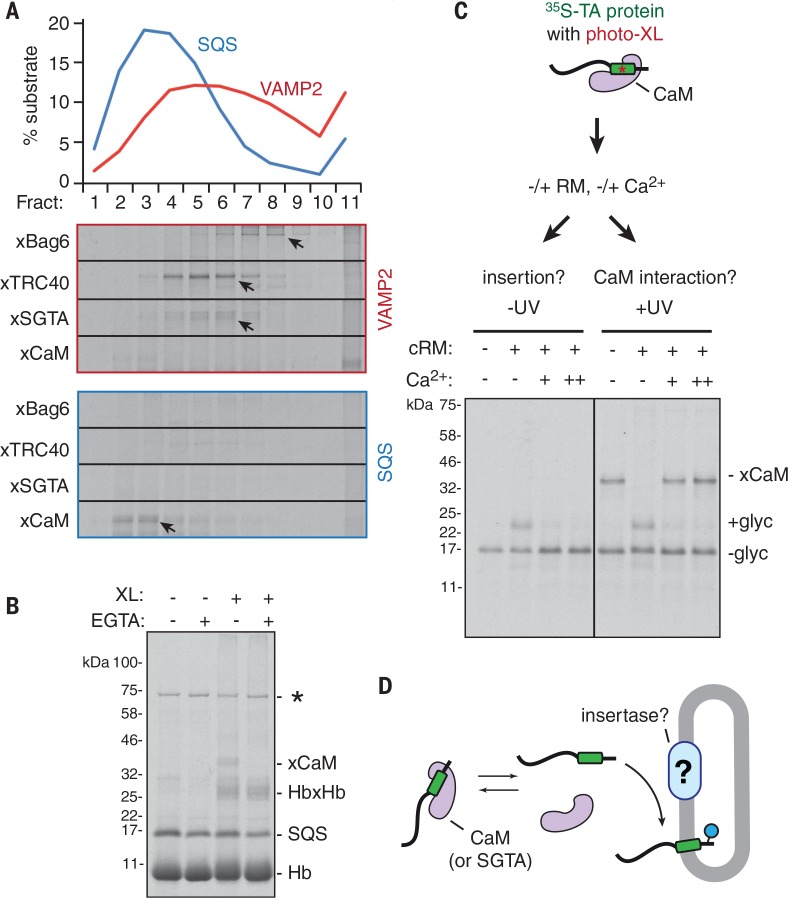
**Identification of cytosolic factors that maintain TA protein insertion competence.** (**A**) ^35^S-methionine–labeled SQS and VAMP2 were translated in native RRL, separated by size on a sucrose gradient, and subjected to chemical cross-linking of each fraction using amine- or sulfhydryl-reactive cross-linker (indicated with an x) (see fig. S5 for full gels). The graph shows the densitometry profiles of each substrate across the gradient, and the individual panels show regions of the cross-linking gels for the indicated interaction partners (verified by immunoprecipitation and mass spectrometry). (**B**) ^35^S-methionine–labeled SQS translated in native RRL was treated with or without 1 mM EGTA before cross-linking and analysis by SDS–polyacrylamide gel electrophoresis and autoradiography. The major SQS cross-linking partner (xCaM) is not seen with EGTA. Hemoglobin (Hb), its intersubunit cross-link (Hb-Hb), and an unspecified translation product (*) are indicated. XL, cross-linker. (**C**) ^35^S-methionine–labeled SQS containing the benzoyl-phenylalanine photo–cross-linker within the TMD was produced as a defined complex with CaM by using the PURE system (protein expression using recombinant elements; see fig. S6). The isolated SQS-CaM complex, prepared in 100 nM Ca^2+^, was incubated with RM in the absence and presence of excess Ca^2+^ (either 0.2 or 0.5 mM) and analyzed directly (left) or irradiated with ultraviolet (UV) light to induce cross-linking before analysis (right). The glycosylated (+ glyc) and CaM–cross-linked (xCaM) products are indicated. (**D**) Schematic of the SQS insertion pathway, with a hypothetical membrane factor indicated with a question mark.

Recombinant CaM was sufficient to prevent aggregation of SQS in a chaperone-free *Escherichia coli*–based translation system assembled from purified translation factors (fig. S6). Addition of ER microsomes to the SQS-CaM complex resulted in SQS insertion at efficiencies similar to that observed in total cytosol (Fig. 2C), whereas SQS synthesized in the absence of CaM was aggregated and not insertion competent (fig. S7). SQS insertion occurred concomitantly with release from CaM as monitored by site-specific photo–crosslinking (Fig. 2C). This suggested an insertion model where dynamic substrate release from CaM [at physiologic Ca^2+^ concentrations in the cytosol (*13*)] transiently provides opportunities for ER engagement before recapture by CaM. In support of this model, insertionwas precluded if the SQSCaM complex was stabilized with superphysiologic concentrations of Ca^2+^ (Fig. 2C and fig. S8A), but did occur across the entire physiologic range of cytosolic free Ca^2+^ (fig. S8B). Furthermore, the unrelated TMD chaperone SGTA, which also associates with substrates dynamically (*12*), behaved similarly to CaM in supporting insertion of SQS in both complete cytosol (fig. S9) and purified systems (fig. S10). By contrast, the VAMP2-SGTA complex is insertion incompetent into ERmicrosomes unless complemented with TRC40 and the Bag6 complex (*12*). Thus, there appears to be a non-TRC pathway tuned to TMDs of moderate to low hydrophobicity. Unlike the highly coordinated TRC targeting system (*2*, *12*), the alternative route can utilize any TMD-shielding factor capable of dynamically releasing substrate for attempts at membrane insertion (Fig. 2D). In native cytosol, the primary factor is CaM(fig. S11), although SGTA can substitute in its absence.

Trypsin sensitivity of the SQS insertion reaction (Fig. 1F) suggested that this critical step is protein mediated. Taking a candidate approach, we considered factors that are conserved across eukaryotes, are abundant, and cause pleiotropic membrane-associated phenotypes when deleted.

In preliminary experiments, we observed no effect on SQS insertion of Sec61 inhibition or knockdown of Sec62 or Sec63, arguing against these possibilities (fig. S12). Although genes of the SRP-independent (SND) targeting pathway are synthetic lethal with TRC pathway mutants in yeast (*14*), appreciable impairment of TA protein insertion was not seen in yeast or mammalian cells lacking SND genes (*14*, *15*). We then considered the ER membrane protein complex (EMC), a widely conserved eight- to ten-subunit complex of unknown function (*16*–*18*) (fig. S13A). The EMC is genetically implicated in many unrelated membrane-associated processes such as quality control, trafficking, protein maturation, and lipid homeostasis (*17*–*22*), but its biochemical activity has been elusive.

Using semipermeabilized cultured cells as the source of ER (fig. S13B), we initially noticed that SQS insertion was partially impaired when the EMC5 subunit of EMCwas depletedwith siRNAs (fig. S13C). Ablation of EMC5 or EMC6 expression by gene editing of osteosarcoma U2OS cells (fig. S14) reduced insertion of SQS, but not VAMP2 (Fig. 3A). This deficiency was rescued by reexpression of EMC5 and EMC6 in the respective knockout cell lines. EMC-dependence was also observed when using ER microsomes isolated from human embryonic kidney (HEK) 293 cells either containing or lacking EMC6 (Fig. 3A). This phenotype was seen regardless ofwhether the substrates were prepared in crude cytosol (Fig. 3A) or provided as defined complexes with CaM (Fig. 3B) or SGTA (fig. S10B).

**Fig. 3:**
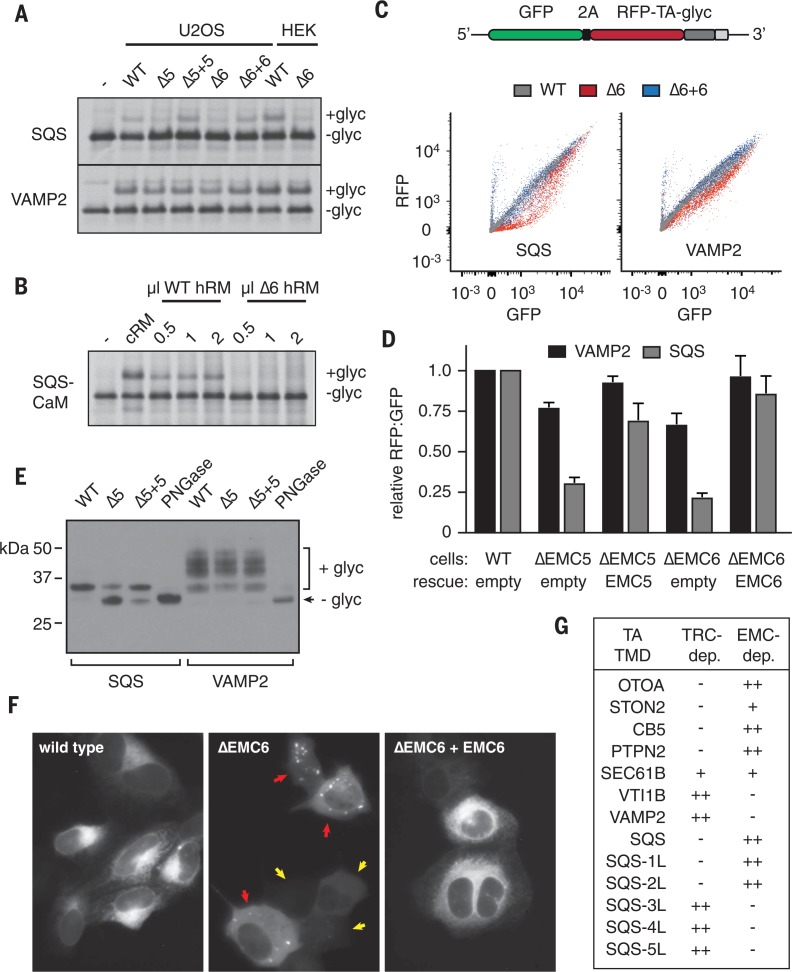
**The EMC is essential for TA protein insertion in vitro and in cells.** (**A**) Semipermeabilized cells (see fig. S13B) fromwild-type (WT) and knockout (D) cells of the indicated cell lines were tested for insertion of SQS and VAMP2 by using the glycosylation assay.The “–” indicates a control reaction lacking semipermeabilized cells. (**B**) The isolated SQS-CaM complex (fig. S6) was tested for insertion into cRM or different amounts of hRM from WTor DEMC6 (D6) HEK293 cell lines. (**C**) Flow cytometry analysis of RFP-SQS and RFP-VAMP2, relative to an internal green fluorescent protein (GFP) expression control (see fig. S15A), in WT (gray), DEMC6 (red), or DEMC6+EMC6 (rescue, blue) cell lines. Although the RFP:GFP ratio remains close to 1 for VAMP2 across a wide range of expression levels in all cell lines, SQS is selectively decreased in DEMC6 cells, especially at low expression levels (see fig. S15B for histograms of these data). 2A, viral 2A peptide. (**D**) Tabulatedmean RFP: GFP ratios for SQS (gray bars) and VAMP2 (black bars) in the indicated cell lines.The results for each construct were normalized to the value in WTcells and depict mean ± SD from three independent experiments. (**E**) Immunoblots for SQS-RFP and VAMP2-RFP in the indicated cell lines. Loadingwas normalized to equivalent amounts of GFP expression as determined by flow cytometry. An aliquot of the WTsample digested with peptide N-glycosidase (PNGase) is shown as a marker for nonglycosylated substrate. Glycosylation of the ER-resident SQS is limited to the core N-glycan, whereas VAMP2 acquires complex glycans because of trafficking through the Golgi. (**F**) Live cell images of GFP-SQS in the indicated cell lines show altered localization in DEMC6 cells. In lowexpressing cells (yellow arrows), the localization is diffusely cytosolic, whereas punctae, presumably representing aggregates, are seen in high-expressing cells (red arrows).VAMP2 was unchanged in its localization in DEMC6 cells (fig. S15C). (**G**) Summary of dependence on either TRC40 (as judged by inhibitory effect of WRB-CC in Fig. 1) or EMC (see fig. S16) for the indicated substrates.

We exploited the fact that noninserted TA proteins are typically degraded (23, 24) to analyze SQS insertion in cells. A red fluorescent protein (RFP)–tagged TA protein construct was varied to contain the TMD of either SQS or VAMP2 and analyzed for expression by flow cytometry,membrane insertion by glycosylation, and cellular location by microscopy. Relative to the nearly unimpaired RFP-VAMP2, RFP-SQS showed reduced expression (Fig. 3, C andD, and fig. S15, A and B), impaired glycosylation (Fig. 3E), and altered localization (Fig. 3F and fig. S15C) selectively in EMC knockout cells. Thus, in vitro and in cells, SQS insertion into the ER is dependent on EMC, the absence of which causes SQSmislocalization, degradation, and aggregation.

Analysis of six other TA proteins and the five SQS TMD mutants showed that each TRC40- independent substrate is strongly EMC dependent (Fig. 3G and fig. S16). Sec61b, a protein of moderate hydrophobicity, showed partial dependence on both EMC and TRC40, identifying the approximate point of overlap between these two pathways. Thus, the TRC- and EMC-dependent pathways aremostly tuned for TMDs of high and low hydrophobicity, respectively, although other features such as TMD length or helicity may also influence pathway choice. The lower hydrophobicity of clients for the EMCpathway presumably explains why a dedicated targeting pathway with constant TMD shielding is not needed, instead relying on temporary release from general TMD binding proteins to engage the membrane.

To determine whether the EMC is sufficient for TA protein insertion, we purified the intact 10-protein complex (Fig. 4A and fig. S17) and optimized conditions for its reconstitution into liposomes. The reconstituted EMC remained fully intact (fig. S18A), with approximately one-third of the complex oriented correctly (fig. S18B). In a protease-protection assay (Fig. 4B), SQS synthesized in native cytosol inserted into EMC proteoliposomes with approximately half of the efficiency observed in native ER microsomes (Fig. 4C). By contrast, VAMP2 insertion, which is efficient into ER microsomes from both wild-type and EMC6 knockout (ΔEMC6) cells, was poor in EMC proteoliposomes. EMC proteoliposomes also supported insertion of the recombinant SQS-CaM complex at near-native levels of insertion relative to ER microsomes (Fig. 4D) when the amount of correctly oriented EMCwasmatched (fig. S18, B and C). As expected, SQS insertion was minimal into liposomes (Fig. 4D) or EMC proteoliposomes pretreated with trypsin (fig. S19).

**Fig. 4:**
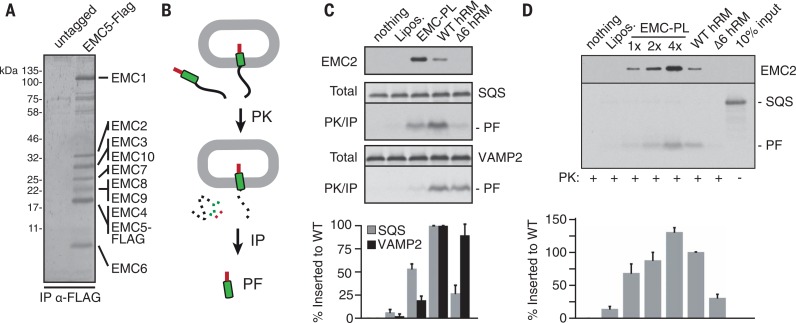
**Reconstitution of EMC-dependent TA protein insertion with purified factors.** (**A**) SYPRO Ruby–stained gel of anti-FLAG (a-FLAG) affinity purification from HEK293 cells expressing untagged or FLAGtagged EMC5. (**B**) Diagram of the protease-protection assay for TA protein insertion using a C-terminal epitope tag (red) to selectively recover the protected fragment (PF) diagnostic of successful insertion. PK, proteinase K; IP, immunoprecipitation. (**C**) Liposomes reconstituted with or without purified EMC were analyzed for insertion of SQS or VAMP2 synthesized in native RRL. For comparison, native ER microsomes (hRM) from WT or DEMC6 HEK293 cells were tested in parallel. Immunoblot for EMC2 indicates the relative amounts of EMC. As shown in fig. S18, roughly onethird of EMC in the proteoliposomes is in the correct orientation. The graph represents four experiments (mean ± SD), normalized to insertion in WT hRM. (**D**) Liposomes reconstituted with a constant amount of lipids and varying amounts of purified EMC were analyzed by protease protection for insertion relative to WT and DEMC6 hRM. The isolated SQS-CaM complex, an aliquot of which is shown in the last lane, was the substrate for these assays. The samples were also immunoblotted for EMC2 to visualize relative EMC amounts. The graph represents four experiments (mean ± SD) normalized to insertion in WT hRM.

The requirement for EMC in microsomes and in cells for SQS insertion, together with SQS insertion into liposomes at near-native efficiencies by purified EMC, rigorously establishes EMC as an ER-resident insertase for moderately hydrophobic TMDs. Bioinformatic analyses indicate that EMC3 is a distant homolog of Get1 (*25*), a subunit of the insertase for the TRC pathway (*26*). Both Get1 and EMC3 seem to have evolved from an ancestral prokaryotic insertase of the YidC family (*25*), apparently having acquired different substrate specificities in the process. The substrates that fail insertion without EMC probably contribute to many of EMC’s reported phenotypes, such as ER stress (*17*), aberrant membrane protein trafficking or degradation (*18*–*21*), altered lipid homeostasis (*22*), or altered viral replication (*27*).

## Supplementary Material

The ER membrane protein complex is a transmembrane domain insertase

## SUPPLEMENTARY MATERIALS

www.sciencemag.org/content/359/6374/470/suppl/DC1

Figs. S1 to S19

References (28–44)
