# Supplementary material for: The ER membrane protein complex is a transmembrane domain insertase
Source: Science. 2017 Dec 14;359:470–3. doi: 10.1126/science.aao3099 (PMC5788257; doi:10.1126/science.aao3099)
Supplement: The ER membrane protein complex is a transmembrane domain insertase [file Science-359-470-s001.pdf]

www.sciencemag.org/cgi/content/full/science.aao3099/DC1

Supplementary Materials for

The ER membrane protein complex is a transmembrane domain insertase

Alina Guna, Norbert Volkmar, John C. Christianson, Ramanujan S. Hegde*

*Corresponding author. Email: rhegde@mrc-lmb.cam.ac.uk

Published 14 December 2017 on *Science* First Release
DOI: 10.1126/science.aao3099

This PDF file includes:

Materials and Methods
Figs. S1 to S19
References

Materials and Methods

Plasmids, antibodies, siRNA, proteins, and reagents

Constructs for expression in rabbit reticulocyte lysate (RRL) were based on the SP64 vector (Promega). Constructs for translation in the PURE system (*28*) were based on the T7-based PURExpress plasmid provided by New England Biolabs. Transmembrane domains (TMDs) of tail anchored (TA) proteins were derived from previous bionformatic analyses (*1*), and included the following human proteins with their respective accession numbers: squalene synthease isoform 1 (SQS; NP_004453.3); otoancorin isoform 2 (OTOA, NP_733764.1); cytochrome b5 isoform 1 (CB5, NP_683725.1); tyrosine-protein phosphatase non-receptor type 2 isoform 1 (PTPN2, NP_002819.1); stonin-2 isoform 1 (STON2, NP_149095.2); vesicle transport through interaction with t-SNAREs homolog 1B (VTI1B, NP_006361.1); Sec61β (SEC61B, NP_006799.1); vesicle associated membrane protein 2 (VAMP2, NP_055047.1). TA protein expression constructs typically contained a C-terminal opsin tag with a glycosylation acceptor site to monitor insertion (*9*) and an N-terminal 3X FLAG tag for affinity purification (see fig S1). Constructs used in all protease protection assays (Fig. 4 and fig. S19), with the exception of fig. S7B, instead had a C-terminal 3F4-tag, which contains two methionines to increase radiolabel incorporation and an option to immunoprecipitate the protected fragment to verify its identity (*29*). Human VCAM1 has been previously described (*30*). All constructs for expression in cultured cells were in the pcDNA5/FRT/TO vector. EMC5 and EMC6 cDNAs were of human origin, and the 3X-FLAG tag in the EMC5-FLAG construct was appended at the C-terminus. GFP-SQS used for microscopy (Fig. 3F) was generated by PCR amplification of the C-terminal region containing the TMD and flanking residues (aa 355-417 of SQS) and fused in-frame downstream of GFP. The dual color reporter for protein degradation (fig. S15A) was based on constructs described previously (*24*), but with the TMD of the desired TA protein appended to the end of the RFP coding region instead of to GFP. Note that the mCherry variant of RFP and the mEGFP variant of GFP were used throughout, but the simpler nomenclature of RFP and GFP are used in the text and figures.

Constructs used for the purification of recombinant proteins included: His-tagged calmodulin in the pRSETA vector (*13*), GST-tagged SGTA in the pGEX-6p1 vector (*12*), and His-tagged human WRB-coiled coil (residues 35 to 101) in the pRSETA vector. Rabbit polyclonal antibodies raised against SGTA, TRC40, GFP, the 3F4 tag and mCherry have previously been described (*3*, *29*, *31*). Additional antibodies were from the following sources: EMC1 (kind gift of Espreafico lab); EMC2 (Proteintech #25443-1-AP); EMC3 (Abcam #ab175537); EMC4 (Abcam #ab123719); EMC5 (Abcam #ab174366); EMC6 (Abcam #ab84902), and tubulin (Sigma #T5168). Pre-designed and validated Silencer Select siRNA from Thermo Fisher were obtained for EMC5 (s41129), SEC62 (s14188), and SEC63 (s22166) knockdowns.

His-tagged calmodulin, GST-tagged SGTA, His-tagged WRB fragment, and His-tagged BpA-RS (for PURE system amber suppression and 'sense codon suppression' in the RRL system) were expressed and purified from BL21(DE3) or BL21(DE3) pLysS *E. coli* cells as previously described (*12*). The CAML peptide (ASQRRAELRRRKLLMNSEQRINRIMGGGWC) and matched R17E mutant were synthesized and purified by Designer BioScience Ltd. Cotransin (CT8) was a generous gift of Jack Taunton and was prepared as previously described (*32*).

TMD hydrophobicity analysis

The substrates were subjected to transmembrane domain prediction using TMHMM (*33*) using an online server (http://www.cbs.dtu.dk/services/TMHMM/) to define the TMD. Relative

hydrophobicity was determined using the transmembrane tendency values for individual amino acids as defined by Zhao and London (*34*), then summing all the values for for the residues in the TMD to arrive at a net hydrophobicity score. It should be noted that although a wide range of scales for determining hydrophobicity exist, there is a very strong correlation between all of them (*35*) and our ranking of relative hydrophobicity between different TA proteins is largely unaffected regardless of the method used.

Mammalian in vitro translation

Translation reactions in RRL, preparation of ER-derived rough microsomes from canine pancreas (cRM) or cultured HEK293 mammalian cells (hRM), and preparation of semi-permeabilized cells, were essentially as described in detail previously (*24*, *36*–*38*) and summarized in brief below. Two RRL-based systems were employed. In cases where we sought to retain the functionality of CaM, a completely native (non-nucleased) RRL was used. This system contains endogenous mRNAs (primarily encoding the ~14 kD globins and a prominent ~70 kD protein) that contribute to background, but has the advantage of retaining native cytosolic conditions, most notably endogenous levels of Ca^{2+}. In some experiments, nucleased RRL was used. Here, $CaCl_2$ is added to 1 mM and the Ca^{2+}-activated nuclease from *S. aureus* is used to digest endogenous mRNA. Then, the nuclease is inactivated by Ca^{2+}-chelation with 2 mM EGTA (*39*). Although this reduces background translation (for comparison, see fig. S6), free Ca^{2+} is estimated to be sub-physiologic [10 nM according to Schoenmakers et al. (*40*)] which renders CaM comparatively inactive for substrate binding. This permits other TMD binding proteins increased access to substrate. The individual figure legends indicate whether native RRL or nucleased RRL was used.

Templates for in vitro transcription (for subsequent translation in RRL) were generated by PCR using a 5' primer which anneals just upstream of the SP6 promoter and a 3' primer that anneals ~200 bp downstream of the stop codon (*36*). Transcription reactions were with SP6 polymerase at 37 °C for 1 hour. The reaction was used directly in a translation reaction without further purification as described (*36*). TA proteins were translated for 20 minutes at 32 °C unless otherwise indicated. For targeting reactions, 1 mM puromycin was added to prevent further protein synthesis, cRM, hRM, or semi-permeabilized cells were added, and reactions were incubated at 32 °C for an additional 15 min. To assay for WRB-coiled coil or CAML peptide inhibition, purified protein or peptide was added at the time of microsome addition. In some reactions, a tripeptide competitive inhibitor of glycosylation (Asn-Tyr-Thr) was added at 50 µM to verify identity of the glycosylated product.

Incorporation of benzoyl-phenylalanine (BpA) into RRL translation products was achieved by the addition of 250 nM BpA and 0.1 mg/ml purified recombinant BpA-RS (*41*) to the translation reaction. BpA-RS is a Tyr-tRNA synthetase from *Methanocaldococcus janaschii* containing mutations that permit its use of BpA instead of Tyr. Because the identity elements of eukaryotic Tyr-tRNA are recognized by the archaeal synthetase (*42*) a subset of Tyr-tRNA in the RRL translation reaction will be charged with BpA instead of Tyr. This allows stochastic incorporation of BpA at Tyr codons. Immediately after the translation reaction, the samples were irradiated on ice with UV light from a UVP B-100 series lamp (UVP LLC) for 15 minutes ~8 cm from the light source. After crosslinking, the samples (typically 20 µl) were layered onto 180 µl of 20% sucrose in PSB and centrifuged at 100,000 rpm for 30 min in a TLA120.1 rotor (Beckman) to remove any incomplete ribosome-associted products and aggregates. The supernatant was then subject to immunoprecipitation of the substrate before SDS-PAGE.

PURE in vitro translation reactions and photo-crosslinking

A modified amber-suppression competent homemade PURE translation system based on previously published methods (*28*) was prepared as previously described (*12*). This system replaces RF1 with total *E. coli* tRNA from a strain over-expressing the amber suppressor tRNA, and contains 50 µg/ml of the purified amber suppressor tRNA synthetase (BpA-RS) and 0.1 mM of the un-natural photo-crosslinking amino acid benzoyl-phenylalanine (BpA). Note that unlike in RRL, BpA-RS is orthogonal in *E. coli*, so BpA is only incorporated at amber codons. Translation reactions were carried out for 30 minutes at 37 °C. Purified SGTA or CaM were added to translation reactions at 12 µM as indicated in the individual figure legends. Reactions with CaM also contained $CaCl_2$ to 100 nM. To isolate the TA-CaM or TA-SGTA complexes, 5 µl translation reactions were diluted to 20 µl with ice cold physiologic salt buffer (PSB: 50 mM HEPES pH 7.4, 100 mM KOAc, 2 mM $MgAc_2$) and size fractionated on a 200 µl 5-25% sucrose gradient in PSB (supplemented with 100 nM $CaCl_2$ in the case of CaM complexes). Centrifugation was for 140 min at 4 °C in a TLS-55 rotor with the slowest acceleration and deceleration settings. Eleven 20 µl fractions were collected from the top, and peak fractions containing TA-chaperone complex were pooled (fractions 2-4 for CaM-SQS and fractions 3-5 for SGTA-CaM) for downstream assays. The concentration of the chaperone in the final insertion assay was between 250 and 500 nM, with the radiolabelled substrate at sub-stoichiometric levels (at least 5-fold lower). Crosslinking analyses in the PURE system were done on ice ~10 cm away from a UVP B-100 series lamp (UVP LLC) for 15 minutes. After crosslinking, protein sample buffer was added directly for SDS-PAGE analysis.

Semi-permeabilized cells and cell-derived microsomes

Semi-permeabilized cells used for targeting reactions were prepared from untreated or siRNA-treated HEK293 cells. The cells were first washed with PBS and cooled on ice before incubation with 100 ng/ml digitonin in 100 mM KAc, 50 mM Tris pH 8.0, 10 mM $MgAc_2$ for 5 minutes on ice. Cells were washed twice with the above buffer lacking digitonin, collected by centrifugation at 12,000 x g for 15 s on a benchtop centrifuge, and re-suspended in 50 mM KAc, 25 mM HEPES, 1 mM $MgAc_2$ on ice to a concentration of ~ $2.5x10^7$ cells per ml. They were kept on ice and used immediately for insertion assays.

Microsomes from HEK293 cells (hRM) were made as previously described (*38*). Briefly, cells were washed three times in PBS and collected by spinning at 200 x g for 5 min. Cells were resuspended in three volumes of ice-cold sucrose buffer (10 mM HEPES, 250 mM sucrose, 2 mM $MgCl_2$) with EDTA-free protease inhibitor cocktail (Roche). Cells were mechanically lysed via passage through a 26-gauge needle. The cell lysate was spun at 3,800 x g at 4 °C for 30 min, and clarified a second time with the same spin conditions. The resulting post-nuclear supernatant was centrifuged at 4 °C at 75,000 x g for 1 h in a TLA100.3 rotor. The resulting microsome pellet was resuspended in microsome buffer (10 mM HEPES, 250 mM sucrose, 1 mM $MgCl_2$, 0.5 mM DTT) and adjusted to an A_{280} value of 75. Insertion assays typically contained 1 µl of hRMs per 10 µl reaction.

Trypsin-inactivation of hRM was with 100 µg/ml trypsin for 15 minutes at 32 °C. Trypsin inhibitor was added to 500 µg/ml on ice, and the membranes were re-isolated by centrifugation through a 200 ul 20% sucrose cushion at 55,000 rpm in a TLA-55 rotor. Membranes were resuspended in microsome buffer with 0.1mg/ml trypsin inhibitor.

Fractionation and chemical crosslinking

The methods closely followed earlier published protocols (*11*, *23*, *29*). In short, 200 µl RRL translation reactions of TA proteins were layered onto a 2 ml 5-25% sucrose gradient in PSB and

centrifuged for 5 hours at 4 °C in a TLS-55 rotor. Eleven 200 µl fractions were collected and put on ice. Aliquots of individual fractions were treated with 250 µM bis-maleimido-hexane (BMH) for 30 minutes on ice for sulfhydryl-mediated crosslinking, or 250 µM di-succinimidyl-suberate (DSS) for 30 min at 22 °C for amine-mediated crosslinking. After crosslinking, protein sample buffer was added directly for SDS-PAGE analysis. In some experiments, the sucrose gradient step was omitted, and the total translation reaction was diluted 10-fold in PSB and subjected to crosslinking as above.

Affinity purification

TA protein affinity purifications were performed with FLAG-tagged TA proteins synthesized in the RRL system (*11*). Immediately following the translation reaction, the sample was chilled on ice and incubated with FLAG-M2 agarose (Sigma) for 1-2 hours at 4 °C. The resin was washed five times with PSB at 4 °C and the bound proteins were eluted with 0.2 mg/ml 3X FLAG peptide in 150 mM NaCl, 50 mM HEPES by incubation with mixing for 30 minutes at 25 °C.

The EMC was purified from EMC5-FLAG expressing T-Rex HEK293 cells that had been induced to express EMC5-FLAG with doxycycline for at least three cell divisions. Cells were washed in PBS, collected by centrifugation, and placed on ice. The cell pellet was solubilized on ice for 30 minutes with 1% digitonin in 200 mM NaCl, 50 mM HEPES, 2 mM $MgAc_2$. Cell lysate was centrifuged at 4 °C for 10 min at maximum speed in a tabletop microcentrifuge. The supernatant was carefully removed and incubated with FLAG-M2 agarose (Sigma) at 4 °C for 1.5 hours. The resin was washed five times with 0.1% digitonin, 200 mM NaCl, 50 mM HEPES. Elution was with 0.2 mg/ml 3X FLAG peptide in 0.25% digitonin, 150 mM NaCl, 50 mM HEPES for 30 minutes at 25 °C with mixing. For reconstitution experiments, the EMC was purified in the identical manner, but using deoxy-BigChap (DBC) rather than digitonin. Identical results were obtained in both of these detergents. To assess complex integrity, 20 µl of the eluted product was layered onto a 200 µl 5-25% sucrose gradient with 100 mM NaCl, 50 mM HEPES, 2 mM $MgAc_2$ and either 0.25% DBC or 0.25% digitonin and spun for 100 minutes at 55,000 rpm in a TLS-55 rotor with slow acceleration and deceleration. Eleven 20 µl fractions were collected and analyzed by SDS-PAGE and Sypro-Ruby staining or immunoblotting.

Proteoliposome reconstitutions and insertion assay

Reconstitution of proteins into liposomes followed minor variations of earlier methods (*4*). Phospholipids were obtained from Avanti Polar Lipids and included phosphatidyl-choline (PC) and phosphatidyl-ethanolamine (PE) from bovine liver, and synthetic 1,2-dioleoyl-*sn*-glycero-3-phosphoethanolamine-N-lissamine rhodamine B (Rh-PE). The standard liposome mixture contained PC:PE:Rh-PE at a mass ratio of 8:1.9:0.1. Rh-PE was used for quantification and to monitor recovery throughout the reconstitution procedure. Lipids were mixed in the above ratios as chloroform stocks, adjusted to 10 mM DTT and dried by centrifugation under vacuum for 16 hours (SpeedVac, Eppendorf). Lipid films were rehydrated to a final concentration of 20 mg/ml in lipid buffer (15% glycerol, 50 mM HEPES pH 7.4) and mixed end over end for 8 hours at 25 °C with occasional vortexing until the mixture was homogeneous. The lipids were diluted with additional lipid buffer and supplemented with deoxy-BigCHAP (DBC) to produce a lipid/DBC mixture containing 2% DBC and 10 mg/ml lipids.

BioBeads-SM2 (BioRad) were prepared by first wetting them with methanol, then washing extensively with distilled water. After all traces of methanol were removed, the beads were adjusted with water so that the settled beads occupied 50% volume. For use in reconstitutions, the BioBeads were dispensed from this 50% slurry in the desired amount, and the excess liquid

was removed by aspiration just before use. The volumes of BioBeads referred to below indicate the packed volume of beads.

Reconstitutions used purified EMC in 0.25% DBC obtained as described above. In initial experiments, we determined the relative concentration of purified EMC compared to the amount in native hRMs from HEK293 cells. This relative concentration was used to gauge the amount needed in the reconstitution reaction to achieve a range of final EMC levels in liposomes spanning the level found in hRMs. Serial dilutions of purified EMC were mixed with a constant amount of lipids and were adjusted so the final buffer concentration was 100 mM NaCl, 25 mM HEPES, 2 mM $MgCl_2$, 0.8% DBC. Control reactions contained the same buffer and detergent conditions, but lacked protein. A standard 100 µl reaction contained 10-40 µl purified EMC, 30 µl of the 10 mg/ml lipid/DBC mixture, and the remaining volume made up with buffer, salts, and detergent. This protein/lipid/detergent mixture was added to 40 µl BioBeads in round bottom 2 ml tubes. The slurry was gently mixed in a thermomixer for 18 hours at 4 °C. The fluid phase was then removed separated and diluted with five volumes of ice-cold water. The proteoliposomes were then sedimented in a TLA120.2 rotor at 70,000 rpm for 30 minutes, and resuspended in 25 µl liposome resuspension buffer (100 mM KAc, 50 mM HEPES pH 7.4, 2 mM $MgAc_2$, 250 mM sucrose, 1 mM DTT).

In early experiments, we found that insertion efficiencies into liposomes and proteoliposomes (PLs) were variable, although EMC-PLs were consistently more active. Subsequent characterization led to the finding that freeze-thaw cycles substantially increase promiscuous insertion into liposomes, but not microsomes. This suggested that the membrane bilayer of liposomes and PLs were more fragile than the native membrane of microsomes. This fragility could be reduced partially (but not eliminated) by inclusion of cholesterol (at 1.2 mg per 10 mg total phospholipids) in the lipid mixture during reconstitution. Thus, when used immediately after preparation, cholesterol at this concentration had no noticeable impact on insertion activity into either liposomes and PLs; however, the cholesterol-containing vesicles were less variable after a freeze-thaw cycle.Thus, for the experiments shown and quantified in the figures, the assays were either performed on freshly prepared vesicles, or once freeze-thawed vesicles that contained cholesterol.

Insertion assays contained 5.5 µl of purified CaM-SQS complex, 2 µl of hRM, 4 µl liposomes, or 4 µl proteoliposomes in a final volume to 10 µl. The reaction was initiated by addition of EGTA to 0.5 mM to chelate excess Ca^{2+} and trigger SQS release from CaM. The insertion reaction proceeded at 32 °C for 20 minutes unless otherwise stated. The samples were transferred to ice, and treated with 0.5 mg/ml proteinase K for 1 hour. The digestion was terminated with 5 mM PMSF for 5 min on ice before being transferred to a 5-fold volume of 1% SDS, 0.1 M Tris pH 8 pre-heated to 100 °C. An additional affinity purification step for the protected TA fragment was performed immediately after. Samples were diluted 10-fold in IP buffer (100 mM NaCl, 50 mM Hepes, pH 7.6, 1% Triton X-100), chilled on ice, and incubated with 2.5 µl 3F4 rabbit polyclonal antiserum and 10 µl Protein A resin for 2 hours at 4 °C. The resin was washed with IP buffer three times and eluted with SDS-PAGE sample buffer before analysis by SDS-PAGE and autoradiography.

Cell culture

U2OS Flp-In TRex cells (kind gift of Mads Gyrd-Hansen, Oxford, UK) and Flp-In TRex 293 cells (Invitrogen) were maintained in DMEM supplemented with 10% fetal calf serum and 2 mM L-glutamine. EMC5 and EMC6 knockout cell lines were generated using the CRISPR/Cas-9

system using previously described protocols (*43*). Cells were transfected with a pSpCas9(BB)-2A-Puro (PX459) plasmid (Addgene) containing sgRNAs targeting EMC5 or EMC6. Cells were then grown for 48 h and treated with puromycin for 72h to select for successfully transfected clones. Single-cell clones were isolated by limiting dilution. Disruption to cell expression was confirmed by western blot of whole cell lysates and additionally by deep sequencing for cells generated in a U2OS background. Multiple independent clones generated with different sgRNAs for each gene were tested for successful knockout and shown to behave similarly in the assays for TA protein insertion. The ΔEMC5 and ΔEMC6 U2OS cells in the figures were made with sgRNAs GCATCATGGCGCCGTCGCTGTGG and GCCGCCTCGCTGATGAACGGCGG, respectively, while the ΔEMC6 HEK293 cells used CCGAGGTCCGGCAATAATCCAGG.

Rescue of the knockout cell lines with the respective expression constructs (in the pcDNA5/FRT/TO vector) was achieved by integration into the FRT locus using the Flp recombinase system as per manufacturer's instructions (Invitrogen). In short, 1×10^6 cells in a 6 cm tissue culture plate were co-transfected with 3 μg of the rescue plasmid and 1 μg of the Flp recombinase expression plasmid (pOG44) using Lipofectamine 2000. Cells stably integrating the gene of interest were selected with 250 μg/ml hygromycin B (U2OS cells) or 100 μg/ml hygromycin B (293 cells) for 7–10 days. Control rescue cells were generated in parallel using the empty pcDNA5/FRT/TO vector and used as the knockout control for comparison in TA protein assays. The cell line expressing EMC5-FLAG was prepared in the same way using Flp-In TRex 293 cells.

Flow cytometry analysis

Cells growing in a 6 cm tissue culture plate were transfected with 250 ng of either GFP-2A-RFP-SQS_{TMD} or GFP-2A-RFP-$VAMP2_{TMD}$. 24 h after transfection, cells were detached with trypsin/EDTA, pelleted and resuspended in ice-cold PBS + 3 mM EDTA, and analysed by flow cytometry using a FACS Canto (BD Biosciences, Franklin Lakes, NJ). The flow cytometry and data analysis (using the FlowJo software package) were essentially as previously described (*24*).

Microscopy

The localization of SQS was visualized in live cells using an expression construct for EGFP-SQS in the pcDNA/FRT/TO vector. After transient transfection and induction of expression for 24 h (100 ng/ml doxycycline), the live cells were visualized at an excitation wavelength of 488 nm using an Olympus CKX41 microscope. The localization of VAMP2 was visualized using confocal microscopy to better discriminate cell surface localization from any potential cytosolic population. Cells growing on 12-mm glass coverslips (Nunc) were transfected with the GFP-2A-RFP-$VAMP2_{TMD}$ construct. After allowing expression for 24 h, the cells were fixed with 4% paraformaldehyde (PFA) and mounted in Fluoromount G (Southern Biotech). Imaging was performed using an LSM 710 confocal microscope (Zeiss).

	TA TMD<br>...FYHSTGGS — MNGTEGPN...	relative hydrophobicity
SQS	SRSHYSPIYLSFVMLLAALSWQYLTTLSQVTED	12.5
OTOA	TRTSSSRSPAGALQSWGLWLGCPLLVLMAKLLW	13.5
CB5	DSSSSWWTNWVIPAISAVAVALMYRLYMAED	15.4
PTPN2	WLYWQPILTKMGFMSVILVGAFVGWRLFFQQNAL	16.5
STON2	ALGSIWLMLPTPFVHPTTLPLLFLLAMLTMFAW	14.2
VTI1B	RKVTTNKLLLSIIILLELAILGGLVYYKFFRSH	22.0
SEC61B	SPGLKVGPVPVLVMSLLFIASVFMLHIWGKYT	21.6
VAMP2	KTGKNLKMMIILGVICAIILIIIIVYFTGSR	27.3
SQS-1L	SRSHYSPIYLSFVMLLAALSWQYLTLLSQVTED	18.1
SQS-2L	SRSHYSPIYLSFVMLLAALSWQYLLLLSQVTED	20.3
SQS-3L	SRSHYSPIYLSFVMLLAALSWLYLLLLSQVTED	23.9
SQS-4L	SRSHYSPIYLSFVMLLAALLWLYLLLLSQVTED	26.3
SQS-5L	SRSHYSPIYLLFVMLLAALLWLYLLLLSQVTED	28.6

Fig. S1. TA proteins used in this study. Sequences of the TMD regions used for analysis and the immediate flanking residues common to all constructs. Red residues indicate mutations made in the SQS TMD to increase its hydrophobicity. These sequences were inserted into the TA protein cassette diagrammed in Fig. 1A. The cassette contains an N-terminal 3X-FLAG tag, the cytosolic domain of the native human TA protein Sec61β, the desired TMD (plus ~5 flanking residues on either side), and a C-terminal domain consisting of the opsin epitope. The opsin tag has a consensus glycosylation sequence that gets efficiently modified in the ER lumen, and therefore serves as a reliable indicator of transmembrane ER insertion. Unless otherwise indicated, all in vitro experiments used this cassette, with the construct referred to simply by the identity of the TMD (e.g., SQS in most contexts refers to this construct containing the TMD from SQS). To the right of each sequence is the "transmembrane tendency" score of Zhao and London (*34*). The region defined as the TMD was determined using the TMHMM algorithm (*33*) and is underlined.

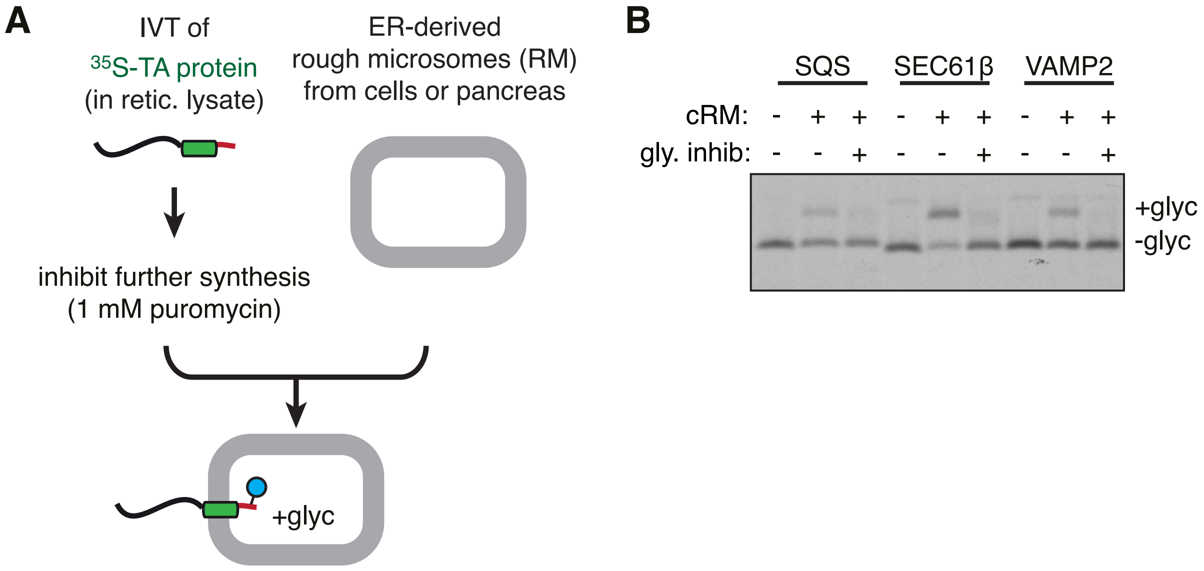

Fig. S2. Glycosylation-based assay for TA protein insertion. (**A**) Basic scheme of the in vitro insertion assay, using glycosylation of the C-terminal opsin tag as the readout. In all insertion assays, the TA protein is ^{35}S-methionine labeled, permitting its detection by autoradiography. Translation was typically performed in reticulocyte lysate, but in later experiments, used the PURE system. (**B**) Example of an insertion assay for SQS, Sec61β, and VAMP2. In each case, samples that contain canine pancrease-derived ER rough microsomes (cRMs) have an addition product (indicated by '+glyc') that represents the glycosylated species. This was verified by its diminishment if a peptide inhibitor of glycosylation was included during the reaction. Note that in the absence of cRMs, a product slightly larger than the glycosylated product is observed. This is a ubiquitinated product that is generated when insertion does not occur, as verified by de-ubiquitinase treatment and ubiquitin pulldowns (data not shown). It is seen faintly in many of the gels in the figures, but usually does not interfere with the interpretation of the results.

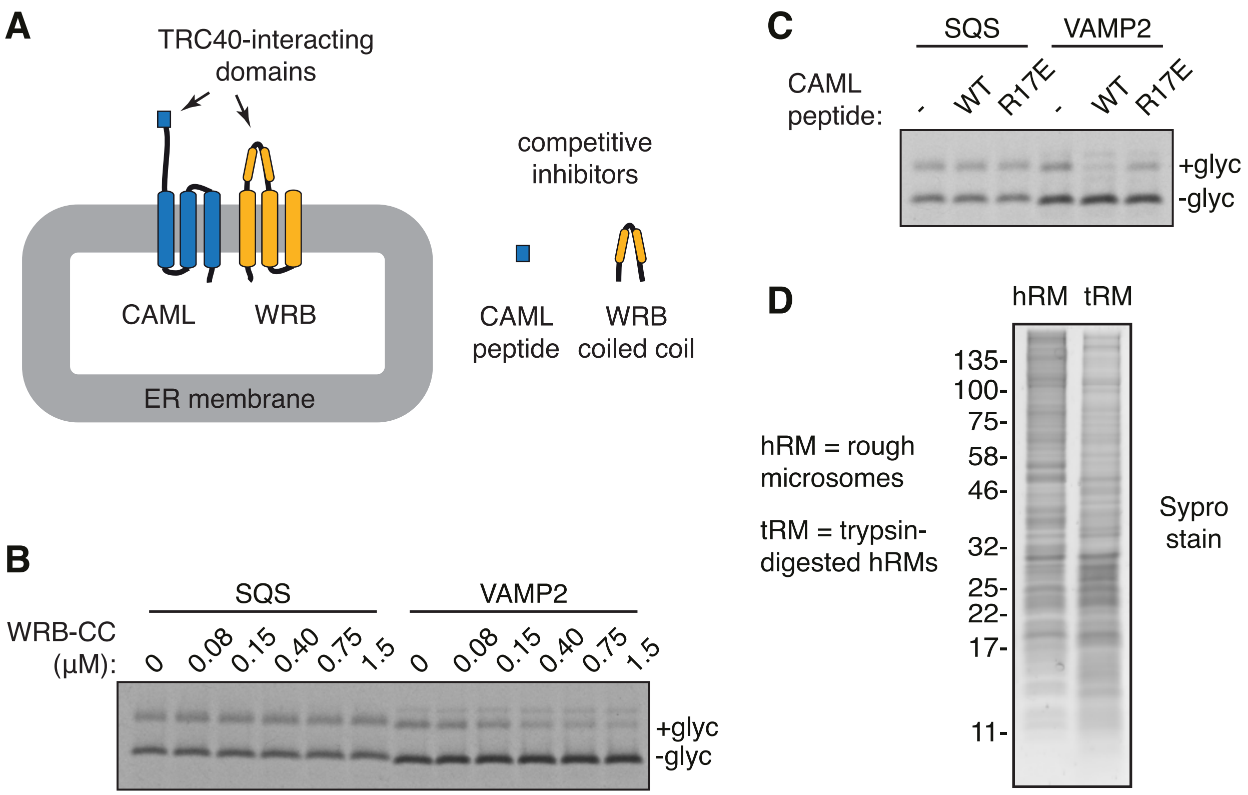

Fig. S3. Characterization of SQS and VAMP2 insertion. (**A**) Diagram of the TRC40 receptor composed of WRB (homologous to yeast Get1) and CAML (homologous to yeast Get2). The domains of each that interact with TRC40 (yeast Get3) are indicated. These fragments can act competitively to inhibit TRC40 interaction with the receptor, and serve as selective probes of the TRC pathway. (**B**) Autoradiograph of an insertion assay for SQS and VAMP2 in the presence of increasing concentrations of the WRB coiled-coil (WRB-CC). VAMP2 is dose-dependently inhibited, while SQS is unaffected. (**C**) Autoradiograph of an insertion assay for SQS and VAMP2 in the presence or absence of the CAML peptide. VAMP2 is inhibited, while SQS is unaffected. A point mutation (R17E) in the peptide that prevents TRC40 interaction is not inhibitory in this assay. (**D**) Sypro Ruby stained gel showing the protein profile of HEK293 cell-derived hRMs that were untreated or digested with trypsin (tRMs) to shave accessible cytosolic proteins.

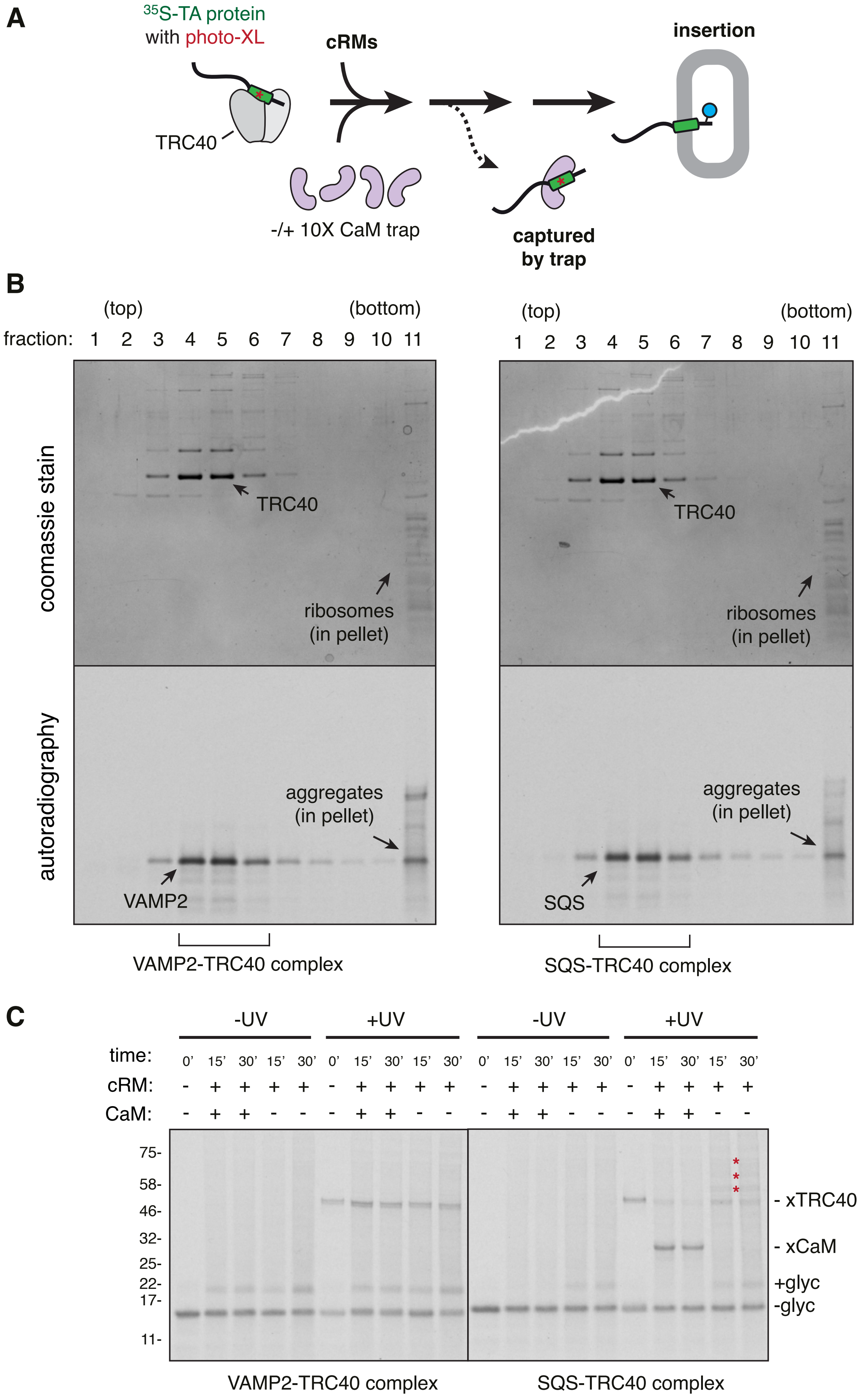
A
35S-TA protein
with photo-XL
TRC40
cRMs
-/+ 10X CaM trap
captured
by trap
insertion
B
(top)
(bottom)
fraction: 1 2 3 4 5 6 7 8 9 10 11
coomassie stain
autoradiography
TRC40
ribosomes
(in pellet)
aggregates
(in pellet)
VAMP2
SQS
VAMP2-TRC40 complex
SQS-TRC40 complex
C
-UV
+UV
time:
cRM:
CaM:
75-
58-
46-
32-
25-
22-
17-
11-
- xTRC40
- xCaM
+glyc
-glyc
VAMP2-TRC40 complex
SQS-TRC40 complex

Fig. S4. TRC40 cannot mediate SQS insertion before dissociation. (**A**) Schematic of the experiment designed to test TRC40-mediated targeting and insertion. The isolated TA-TRC40 complex prepared in the PURE system is presented to cRMs in the presence of a 10-fold excess of Ca^{2+}-CaM. In the presence of excess Ca^{2+}, CaM serves as a trap for exposed TMDs and effectively precludes additional insertion attempts. Thus, if the TA-TRC40 complex is delivered to its receptor and inserted, the CaM trap is inert and never sees the substrate. By contrast, dissociation of TA protein from TRC40 before membrane delivery results in capture by the CaM trap. Interactions between TA protein and TRC40 or CaM can be monitored in this experiment via a photo-crosslinker incorporated into the TMD o the TA protein. (**B**) SQS and VAMP2 were translated in an amber-suppressor competent PURE system (see Methods) supplemented with 12 μM recombinant zebrafish TRC40. An amber codon in the center of each TMD was suppressed with the benzoyl-phenylalanine (BpF) photo-crosslinking residue. Analysis of the products on a sucrose gradient shows that SQS and VAMP2 co-fractionate with TRC40 in fractions 3-6. In the absence of TRC40, both of these proteins normally aggregate quantitatively. Thus, fractions 3-6 represent TA-TRC40 complexes, and were pooled for use in subsequent insertion assays. The TRC40 concentration in this pooled fraction is 750 nM, and becomes diluted to less than ~100 nM in the insertion assay. (**C**) The TA-TRC40 complexes from panel B were added to cRMs in the presence or absence of 1 μM CaM and 1 mM Ca^{2+}. After 15 min or 30 min of incubation at 32 °C, the samples were either analyzed directly or subjected to UV irradiation on ice before analysis. The positions of the glycosylated (+glyc), TRC40-crosslinked (xTRC40) and CaM-crosslinked (xCaM) products are indicated. Note that VAMP2 inserts comparably well regardless of the CaM trap, and no crosslinking to CaM is observed. Thus, the fate of VAMP2-TRC40 is either to remain on TRC40, consistent with its very slow off-rate (*12*), or insert into the ER via its WRB/CAML receptor complex (as shown in fig. S3). By contrast, the SQS-TRC40 complex completely fails insertion in the presence of the CaM trap, and is observed to release from TRC40 and bind to CaM. This indicates that SQS dissociates from TRC40 before its successful insertion. Of note, in the absence of the CaM trap, SQS inserts into cRMs. This shows that upon dissociation from TRC40, the released SQS has a route into the membrane, which later experiments showed was due to EMC-mediated insertion. In this circumstance, the non-inserted SQS also seems to make a heterogeneous set of non-specific interactions (red asterisks) in the absence of any suitable chaperone for this TMD.

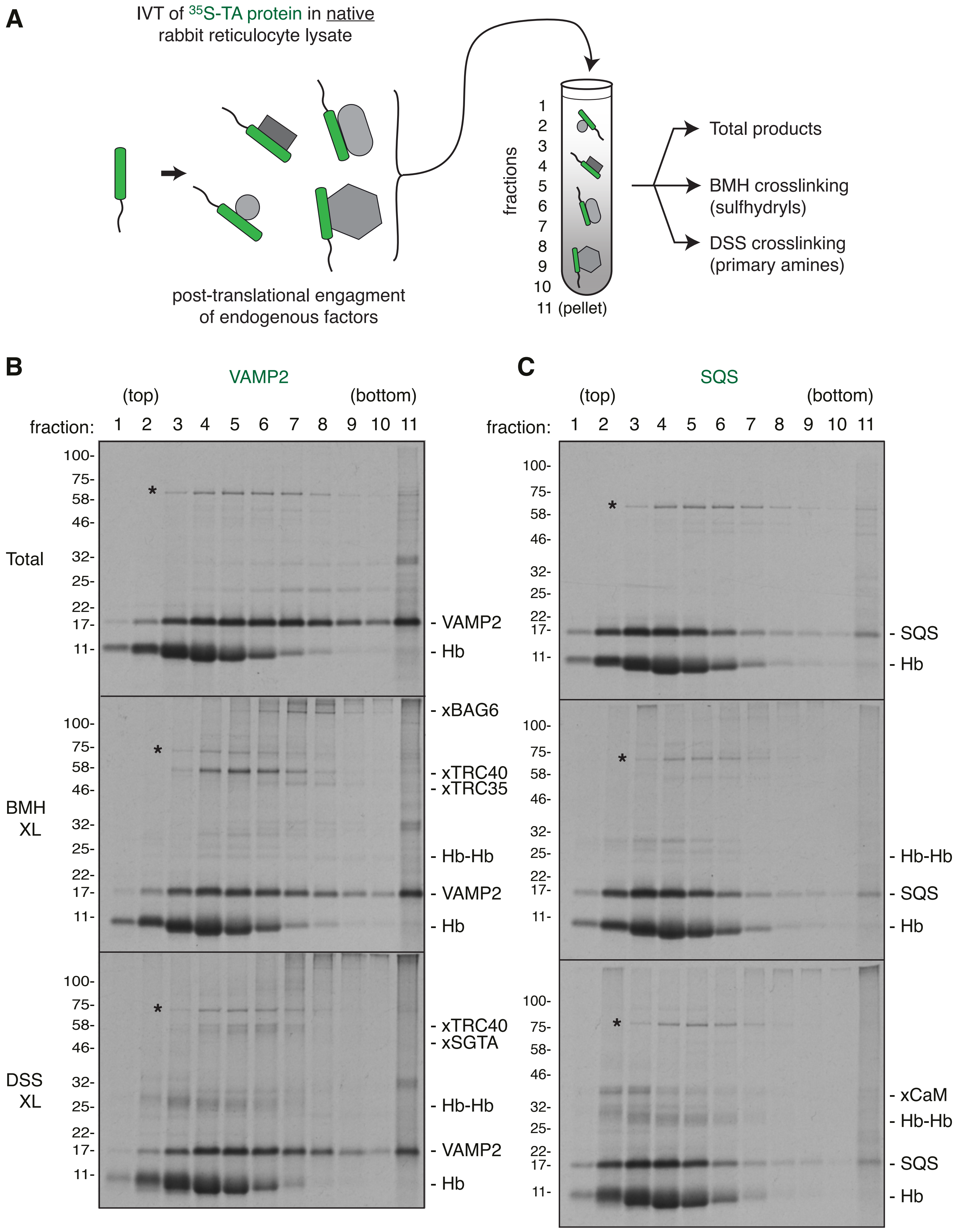

A
IVT of 35S-TA protein in native rabbit reticulocyte lysate
post-translational engagment of endogenous factors
fractions
1
2
3
4
5
6
7
8
9
10
11 (pellet)
Total products
BMH crosslinking (sulfhydryls)
DSS crosslinking (primary amines)
B
VAMP2
(top)
(bottom)
fraction: 1 2 3 4 5 6 7 8 9 10 11
Total
BMH XL
DSS XL
100-
75-
58-
46-
32-
25-
22-
17-
11-
*
- VAMP2
- Hb
- xBAG6
- xTRC40
- xTRC35
- Hb-Hb
- xSGTA
C
SQS
- SQS
- xCaM

Fig. S5. Analysis of interactions by SQS and VAMP2 in native reticulocyte lysate. (**A**) Schematic of the experiment used to analyze cytosolic interactions made by SQS and VAMP2. (**B**, **C**) The products of VAMP2 (panel B) and SQS (panel C) translation in native RRL were separated by sucrose gradient sedimentation and analyzed directly or after chemical crosslinking with bis-maleimido-hexane (BMH) or di-succinimidyl-suberate (DSS). The translation products and their crosslinks were visualized by autoradiography. The primary VAMP2 and SQS translation products are indicated on each gel, as are the translation products of hemoglobin (Hb) and an abundant 70 kD reticulocyte protein. Crosslinks of VAMP2 to Bag6, TRC40, SGTA, and TRC35 are indicated, and have been characterized extensively in earlier work (*11*, *12*, *29*). These crosslinks were extremely weak or non-existent for SQS. Instead, SQS crosslinked to a 20 kD product only with DSS. Affinity purification of large-scale SQS translation reactions identified this interacting partner as CaM. This assignment is consistent with its inability to crosslink via BMH (as CaM has no cysteines), and loss of crosslinking in the presence of the Ca^{2+}-chelator EGTA (Fig. 2B). Thus, in native RRL, the SQS and VAMP2 TMDs make distinct interactions.

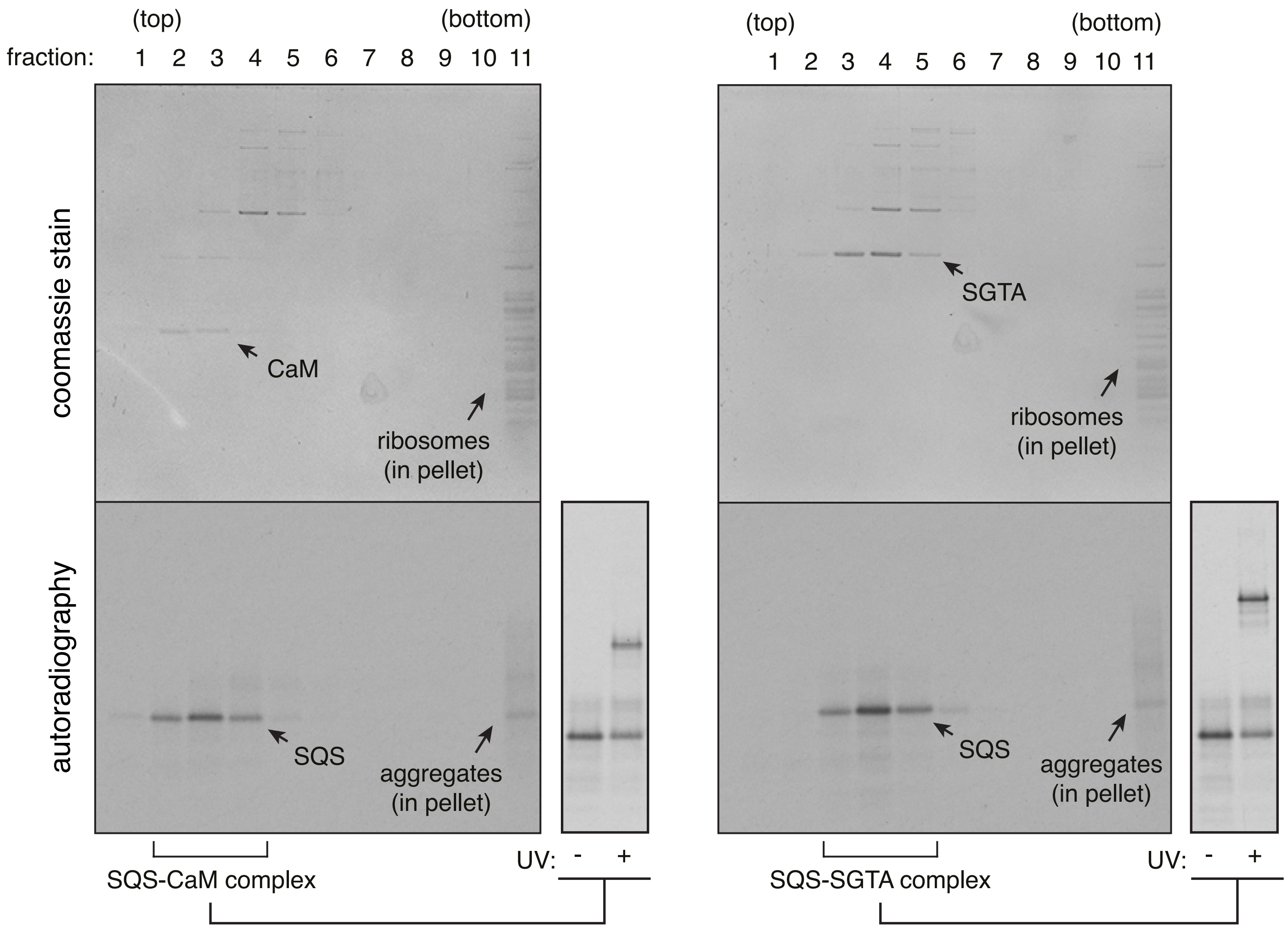

Fig. S6. Preparation of SQS-CaM and SQS-SGTA complexes in the PURE system. SQS was translated in an amber-suppressor competent PURE system (see Methods) supplemented with 12 μM recombinant human CaM or SGTA. An amber codon in the center of the TMD was suppressed with the benzoyl-phenylalanine (BpF) photo-crosslinking residue. Analysis of the products on a sucrose gradient shows that SQS co-fractionates with CaM and SGTA in fractions 2-4 and 3-5, respectively. In the absence of any chaperone, SQS aggregates quantitatively (see fig. S7). Thus, the soluble fractions represent SQS-CaM and SQS-SGTA complexes, and were pooled for use in subsequent insertion assays. The chaperone concentration in this pooled fraction is ~ 1 μM, and becomes diluted to ~200-500 nM in the insertion assay. UV irradiation of the pooled peak fraction verifies that the SQS contains the photo-crosslinking residue and is in a complex with either CaM or SGTA.

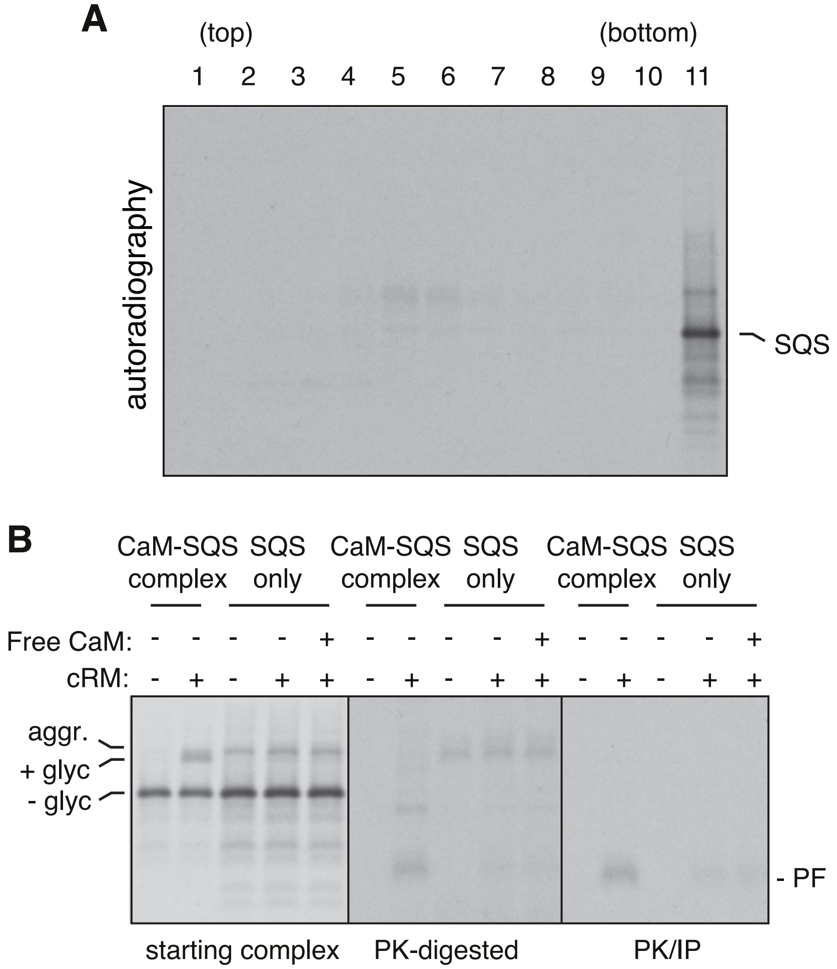

Fig. S7. SQS is not soluble or insertion competent in the PURE system without chaperones. (**A**) SQS was translated in the PURE system (see Methods) in the absence of any added chaperones. Analysis of the products on a sucrose gradient shows that nearly all SQS migrates at the bottom, indicative of aggregation. This contrasts with the behavior of SQS in the presence of a TMD-binding chaperone such as CaM, SGTA, or TRC40 (see fig. S4 and S6 for comparison). A small amount of incompletely aggregated SQS is visible on longer exposures of the autoradiograph. (**B**) SQS was translated in the PURE system containing or lacking CaM ("CaM-SQS complex" and "SQS only", respectively). These total translation reactions were incubated without or with canine pancreas rough microsomes (cRM). In one reaction, the "SQS only" sample was supplemented with CaM post-translationally prior to incubation with RM. After this incubation, the samples were either analyzed directly ("starting complex") or treated with proteinase K (PK). The PK-digested samples were analyzed directly (middle panel) or subjected to immunoprecipitation using antibodies against the C-terminal Opsin tag (right panel). Note that in the starting samples, the CaM-SQS complex promotes insertion (as indicated by appearance of a glycosylated band). A slightly higher molecular weight species apparently represents SDS-resistant aggregated SQS. This aggregated product is partially resistant to protease digestion. Only the C-terminal protected fragment (PF) indicative of successful insertion is immunoprecipitated with the Opsin antibody. The small amount of insertion seen in the "SQS only" sample can be explained by the small amount that escapes aggregation during the initial translation reaction.

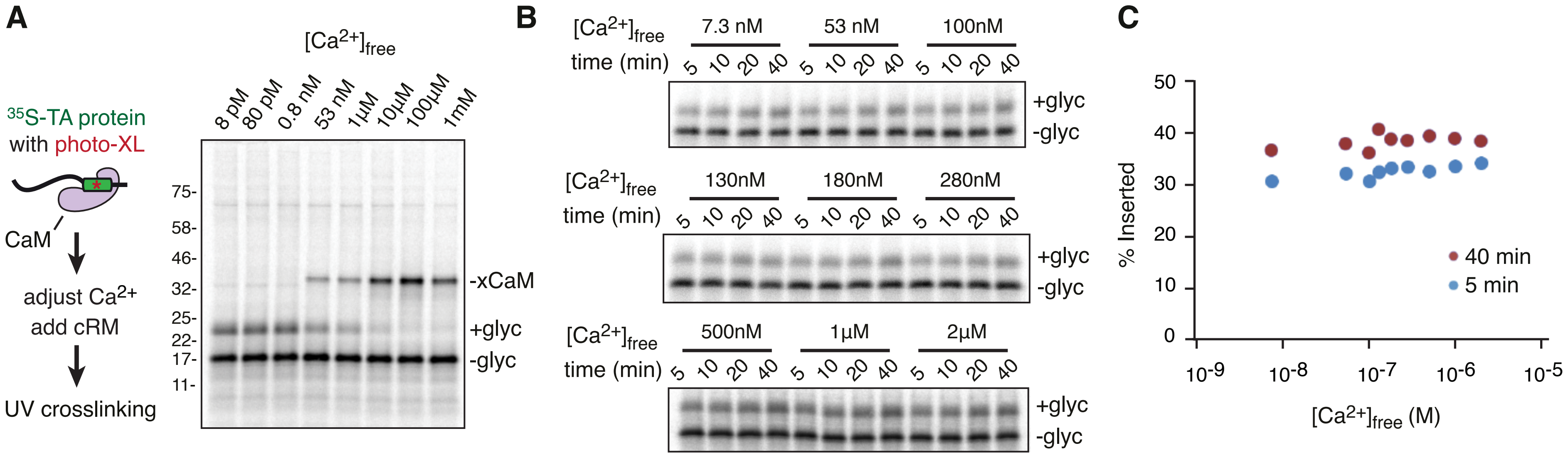

Fig. S8. Calmodulin dynamically interacts with substrates at physiological Ca^{2+} levels. **(A)** CaM-SQS complexes were prepared in the PURE translation system in 100 nM Ca^{2+} and isolated from the peak fractions of a sucrose gradient as shown in fig. S6. The complex was incubated with cRM for 20 min at the indicated concentrations of free Ca^{2+}. To lower the Ca^{2+} below 100 nM, EGTA was added to the appropriate concentration (*40*). Samples were then immediately removed to ice and UV irradiated to allow for visualisation of SQS still in complex with CaM. At high Ca^{2+}, the majority of SQS stays bound to CaM, thereby precluding insertion. **(B)** CaM-SQS complexes were assayed for insertion into cRM within a narrower range of Ca^{2+}, with samples collected at the indicated time points. Resting Ca^{2+} in the cytosol is thought to be ~100 nM. During physiologic stimuli, cytosolic Ca^{2+} is thought to rise to ~1000 nM. **(C)** Quantification of insertion reactions from panel B at 5 and 40 minutes. Note that insertion is rapid, being nearly complete at 5 min. Insertion efficiency is comparable within this physiological range of cytosolic Ca^{2+}, but can be increased slightly further if Ca^{2+} is dropped to sub-nanomolar levels at the same time as microsomes are added (e.g., 0.8 nM or lower, as in panel A).

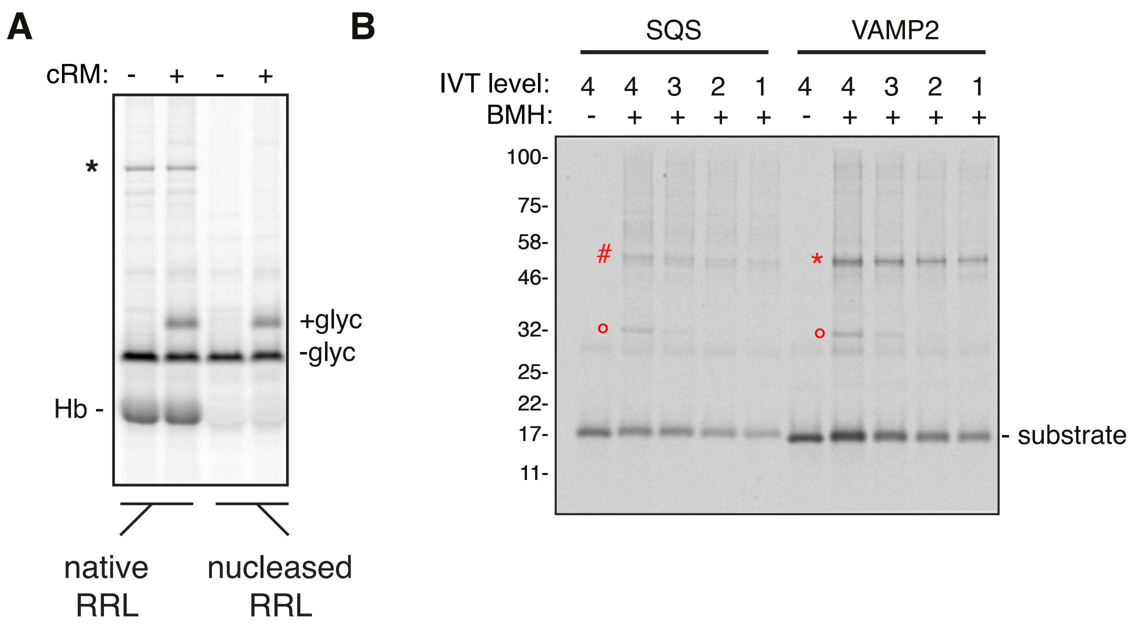

Fig. S9. Analysis of SQS interactions in the absence of CaM. (**A**) SQS was translated in native RRL or the standard nuclease-treated RRL, then incubated with cRM from canine pancreas as indicated. In both cases, SQS was inserted into the membrane with comparable efficiency. Note that hemoglobin (Hb) and the 70 kD product are not translated in nucleased treated RRL. (**B**) SQS and VAMP2 were translated at varying levels of expression in nucleased RRL, after which the lysate was subjected to crosslinking with BMH. All of the samples were immunoprecipitated using the FLAG tag on the substrate and analyzed by SDS-PAGE and autoradiography. At limiting amounts of translation, the primary interaction product is preferentially observed, as there is minimal saturation of any cellular factors by the substrate. For VAMP2, this is TRC40 (indicated by '*'). SQS also interacts with a similar sized product ('#'), but which proved to be SGTA as identified by immunoprecipitation and mass spectrometry of large-scale translation reactions. This interaction was less prominent in native RRL, where CaM is the primary interaction partner (fig. S5, S11). Thus, in the absence of CaM, a major interaction partner for SQS is SGTA. It is worth noting that SQS is also seen to interact with Bag6 and UBQLNs at higher translation levels, presumably for the purpose of degradation in case of failed insertion (*23*, *24*). Furthermore, when interactions are assessed by photo-crosslinking in undiluted translation reactions (fig. S11), TRC40 is also observed. This interaction is apparently dynamic and easily lost upon dilution or immunoprecipitation (e.g., Fig. 1B), explaining why it does not effectively mediate targeting and insertion of SQS (e.g., Fig. 1C and fig. S4). The crosslink indicated by the open circle appears to be substrate self-association seen only at higher translation levels.

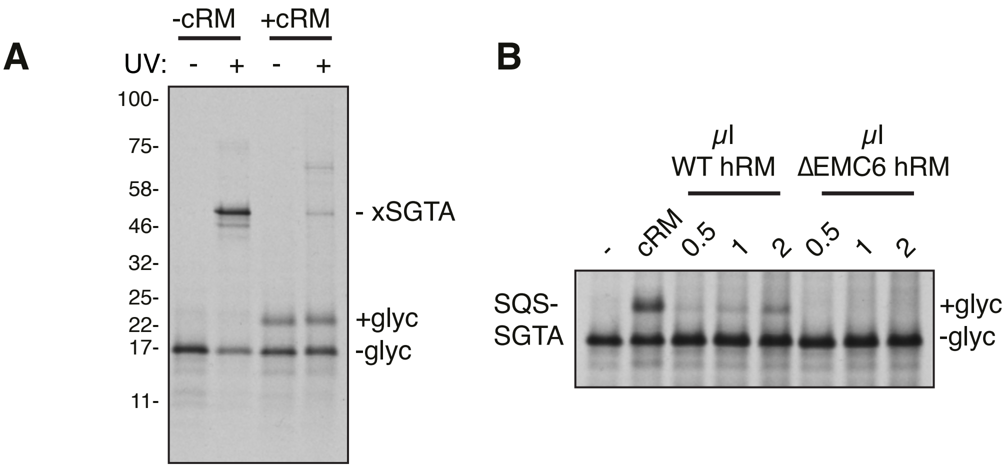

Fig. S10. The SQS-SGTA complex is competent for ER insertion. (**A**) The SQS-SGTA complex prepared in the PURE system (see fig. S6) was incubated without or with canine pancreas RMs (cRM). After the incubation, the samples were divided and one half was subjected to UV irradiation to detect interactions with the TMD of SQS. Insertion was observed as judged by glycosylation (+glyc), and was similar in efficiency as seen for SQS translated in native and nucleased RRL (compare to fig. S9A). Furthermore, insertion is accompanied by release from SGTA. Note that the presence of excess Ca^{2+} had no effect on insertion mediated by the SQS-SGTA complex, in contrast to the inhibitory effect seen with the SQS-CaM complex (Fig. 2C and fig. S8A). (**B**) The SQS-SGTA complex prepared in the PURE system was tested for insertion into microsomes from canine pancreas (cRM) or different amounts of hRM from wild type (WT) or ΔEMC6 HEK293 cells. As seen for the SQS-CaM complex, the SQS-SGTA complex shows EMC-dependent insertion. Note that pancreas-derived microsomes typically show higher overall efficiencies of protein translocation and insertion, presumably because pancreatic ER is more highly enriched in biosynthetic machinery and is a more pure ER preparation.

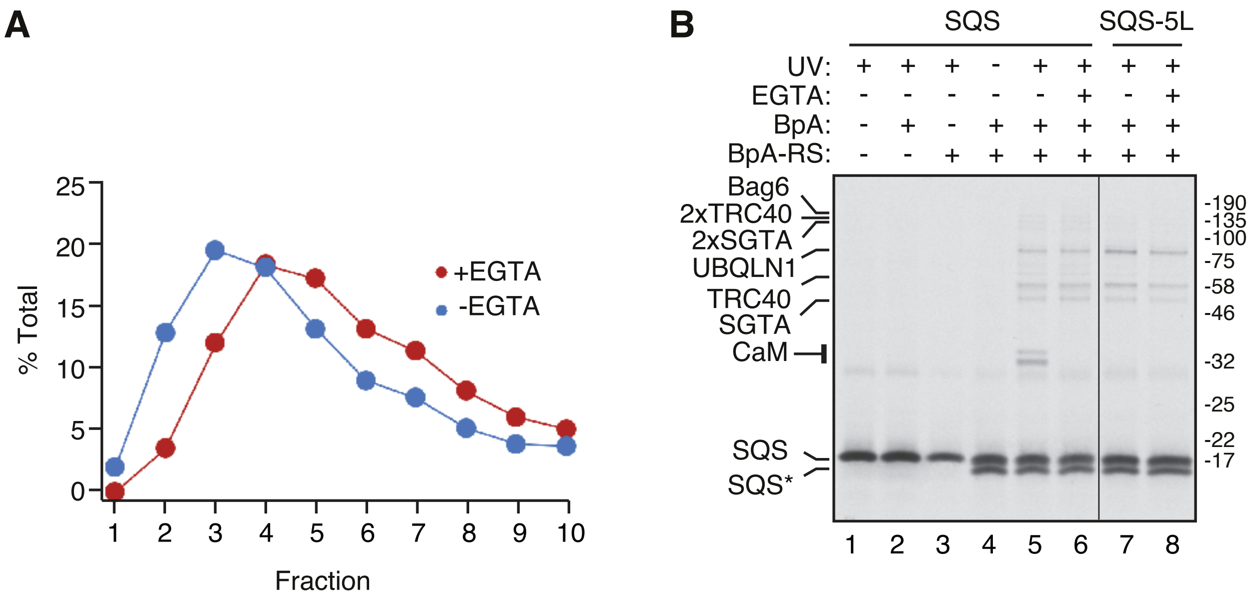

Fig. S11. Calmodulin is the primary interaction partner for the SQS TMD. (**A**) SQS was translated in native RRL in the absence or presence of 1 mM EGTA to chelate endogenous Ca^{2+}. The samples were then separated by size on a 5-25% sucrose gradient, and SQS levels in each fraction were quantified by phosphorimaging. The percent of total SQS in each fraction is plotted. For reference, the peak of native hemoglobin tetramer (60 kD) is in fraction 4. Nearly half of SQS migrates in fractions 1-3, indicating a native size less than 60 kD. Importantly, this population shifts when EGTA is included, indicating that it represents a complex with Calmodulin, as verified by separate crosslinking assays (fig. S5 and fig. S11B). Thus, by this analysis, CaM is one of the primary interaction partners for SQS.

(**B**) SQS or SQS-5L was translated in native RRL supplemented with the photo-reactive crosslinker benzoyl-phenylalanine (BpA) and BpA tRNA synthetase (BpA-RS). BpA-RS will charge BpA onto Tyr-tRNA, thereby competing with endogenous Tyr tRNA synthetase. This will result in BpA incorporation at Tyr codons, three of which are in the TMD of SQS (see fig. S1). Control reactions omitted BpA or BpA-RS, or included 1 mM EGTA in the translation reaction to chelate endogenous Ca^{2+}, as indicated. Following translation, the entire undiluted reaction was irradiated with UV light to induce crosslinking as indicated. Any aggregates were removed by centrifugation and SQS was recovered by anti-FLAG immunoprecipitation. Note that incorporation of BpA results in slightly faster migration of the substrate (SQS*). Inclusion of BpA-RS without BpA (lane 3) results in lower translation, presumably becuase BpA-RS competes for Tyr-tRNA but does not charge it. The major crosslinking partners are indicated, and were verified by immunoprecipitation (not shown), or sensitivity to Ca2+ chelation in the case of CaM. Crosslinking to two molecules of SGTA or TRC40 occurs because these factors bind substrate as dimers.

Phosphorimager quantification of lane 5 (using lane 4 as background) indicate that the visible SQS crosslinking products comprise 45% CaM, 9.7% (SGTA), 12.2% (TRC40), 12.2% (Ubiquilin1), and 1.1% (Bag6) of total crosslinked products in the entire lane. The only unaccounted crosslinking bands that are visible (between the TRC40 and Ubiquilin1 bands) represent 2.9% and 3% of all crosslinking products. The remaining 14.2% of signal in the lane could not be attributed to any discernable band, and presumably represents non-specific heterogeneous crosslinking products. This indicates that: (i) CaM is the major TMD interaction partner in complete lysate; (ii) CaM is specific to SQS relative to SQS-5L; (iii) no unaccounted TMD interaction product that might represent an unidentified targeting factor interacts with more than 3% of substrate.

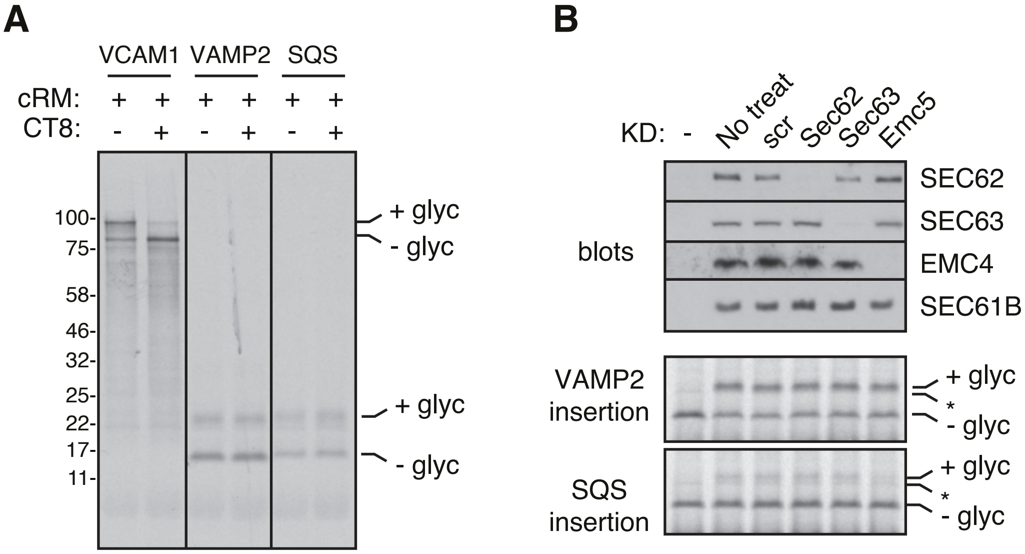

Fig. S12. Sec61/Sec62/Sec63 are not involved in SQS insertion. (A) VCAM1, VAMP2 and SQS were analyzed for translocation or insertion into cRMs without and with 1μM cotransin (CT8), an inhibitor of the Sec61 translocon (*30, 32*). VCAM1 was tested co-translationally, while VAMP2 and SQS were tested post-translationally. In each case, nucleased RRL was used for translation, and translocation was assayed by glycosylation. VCAM1 insertion is clearly impaired in its translocation by CT8 as indicated by a decrease in the glycosylated product. However, VAMP2 and SQS are unimpaired, suggesting that they do not use the Sec61 channel for insertion. **(B)** HEK293 cells were treated with control, SEC62, SEC63, or EMC5 siRNAs for 72 hours. The cells were semi-permeabilized using digitonin (see fig. S13B), and used in an insertion assay for SQS and VAMP2 translated in RRL. Effective knockdown of Sec62 and Sec63 were verified by immunoblotting, while EMC5 knockdown was confirmed by probing levels of EMC4, whose expression depends on an intact EMC (see fig. S14). Insertion was assayed by monitoring glycosylation. A non-specific product (asterisk) is observed just below the glycosylated product in all lanes. Note that neither VAMP2 nor SQS insertion are affected by knockdown of Sec62 or Sec63; however, SQS insertion is noticably impaired in the EMC5 knockdown cells (further characterized in fig. S13).

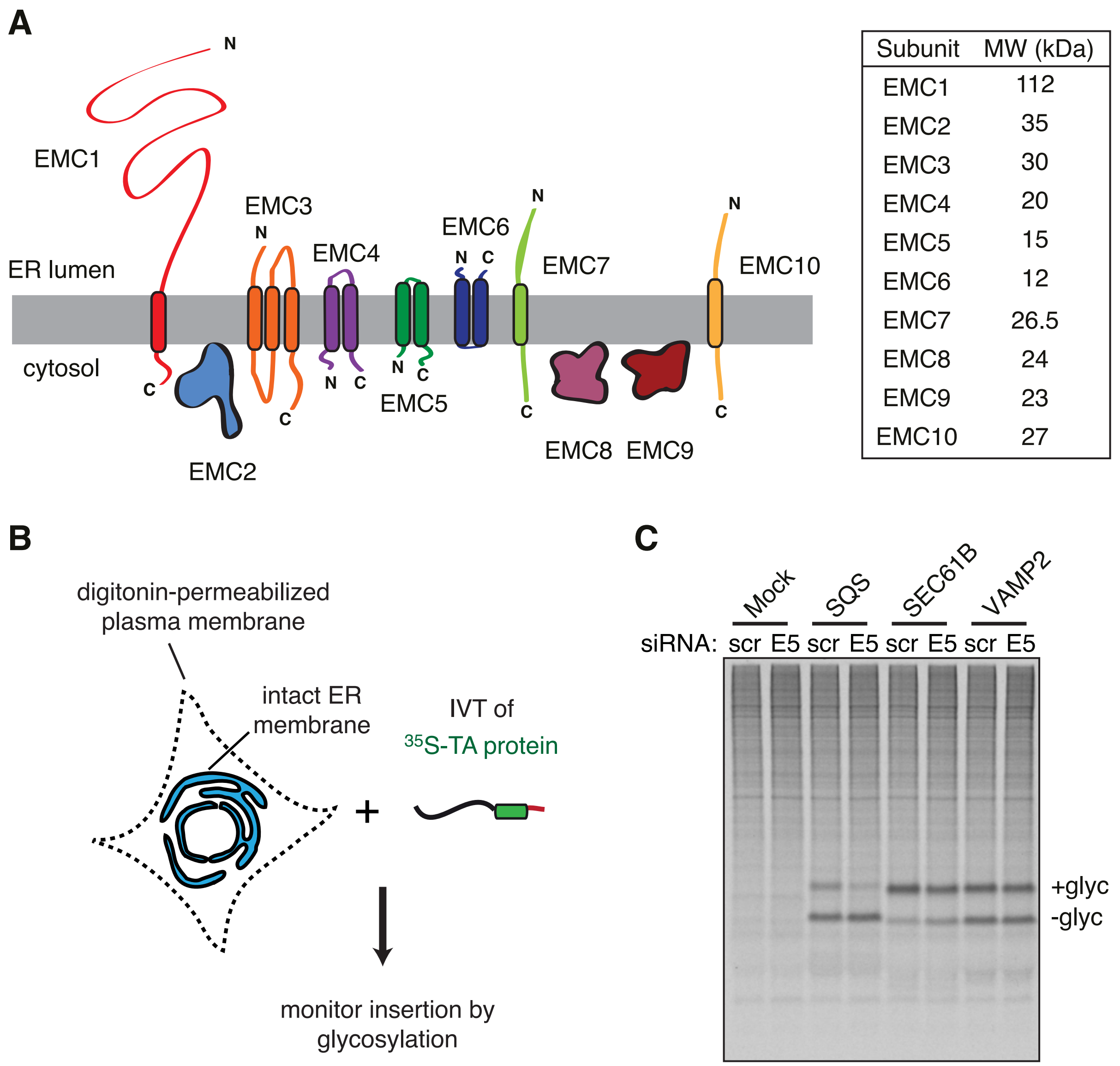

Fig. S13. EMC5 knockdown impairs SQS insertion. (**A**) Schematic diagram of the ten subunits of mammalian EMC and their approximate molecular weights. Seven of the subunits are predicted to be integral membrane proteins, while the other three are thought to be peripherally associated. (**B**) Diagram of the insertion assay using semi-permeabilized cells. (C) HEK293 cells were treated with control or EMC5 siRNAs for 72 hours, and used in an insertion assay for SQS, Sec61β, and VAMP2 as depicted in panel B. Near-complete knockdown of EMC5 was verified by immunoblotting (not shown). Note that insertion of SQS is noticeably impaired, while little or no effect was observed for either Sec61β, and VAMP2. Additional experiments showed that co-translational substrates such as pre-prolactin or prion protein were unaffected by EMC5 knockdown. In this particular experiment, the translation reaction was not treated with puromycin before addition of semi-permeabilized cells, resulting in some translation of endogenous cellular mRNAs observed in all of the lanes.

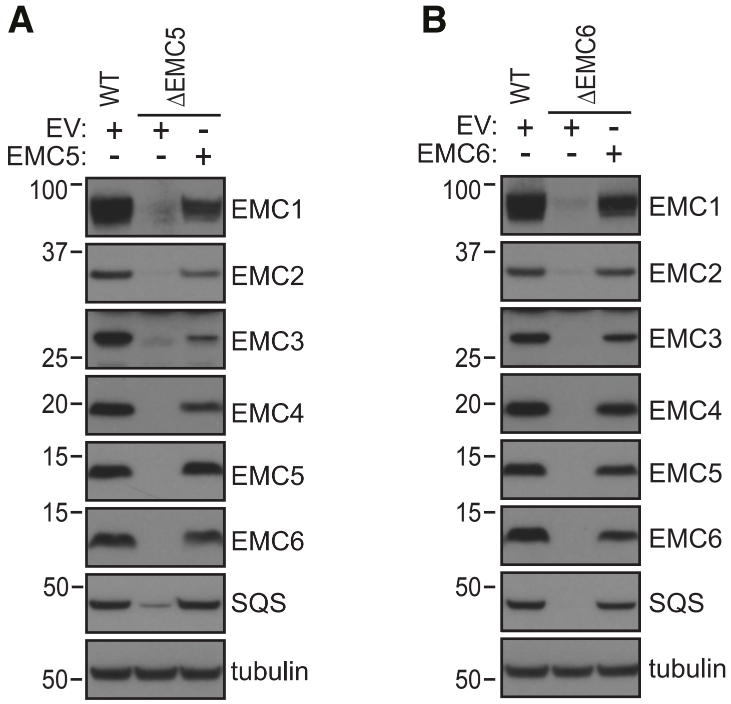

Fig. S14. Characterization of EMC KO and rescue cell lines. The Flp-in Tet-on U2OS cell line was subjected to CRISPR/Cas9-mediated gene editing to disrupt either EMC5 (panel A) or EMC6 (panel B). The resulting cell lines, verified to be knockouts by immunoblotting, were rescued by re-introducing doxycycline-inducible expression constructs for EMC5 or EMC6 into the FRT site. As a control, the empty vector (EV) was introduced into the FRT site as indicated. Shown is an immunoblot for several of the EMC subunits, tubulin, and endogenous SQS in the parental, knockout, and rescue cell lines. Note that knockout of EMC5 or EMC6 strongly disrupts the remainder of the complex, while re-expression restores expression of all subunits to almost normal levels.

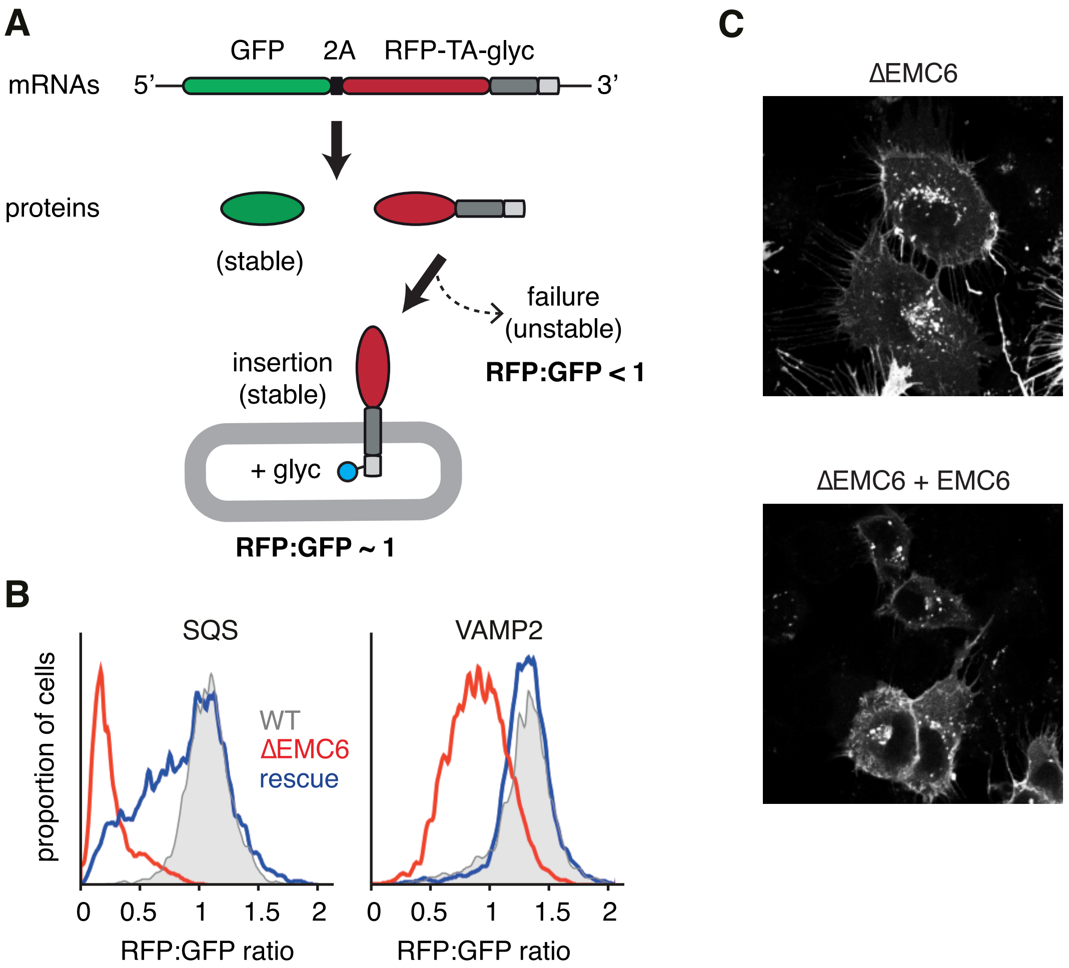

Fig. S15. Characterization of VAMP2 localization in EMC knockout cells. (**A**) Diagram depicting the construct for dual-color analysis of TA protein insertion versus degradation. The viral 2A sequence results in the skipping of a peptide bond during translation, resulting in two separate translation products (*44*). In this case, the GFP serves as an expression control against which the level of RFP-TA can be normalized. If the RFP-TA protein is inserted into the ER, it is relatively stable with a long half-life compared to the situation where insertion fails (*23*, *24*). Thus, a lowering of the RFP:GFP ratio would indicate destabilization of RFP, which could indicate failed insertion. (**B**) Histogram of the RFP:GFP ratio for the SQS and VAMP2 constructs in the indicated cell lines. Relative to wild type cells, the peak of the RFP:GFP ratio is ~5-fold lower for SQS in cells lacking EMC6, and this is mostly rescued by re-expression of EMC6. In the case of VAMP2, the effects are more modest, with the ratio decreasing by less than 2-fold in ΔEMC6 cells. Given that we see no detectable deficiency in VAMP2 insertion by in vitro assays, the small effect in cells might be due to an indirect effect (e.g., on VAMP trafficking or post-insertion turnover) or more likely, a consequence of TRC pathway saturation when the EMC pathway is unavailable. Similar effects were seen when EMC5 was disrupted (Fig. 3D). (**C**) The localization of RFP-VAMP2 was visualized in ΔEMC6 and rescue cells after fixation. No obvious differences in localization were observed across a wide range of expression levels. This contrasts with SQS, whose localization is markedly altered in ΔEMC6 cells (Fig. 3F). A similar SQS-specific effect was observed in ΔEMC5 cells (not shown).

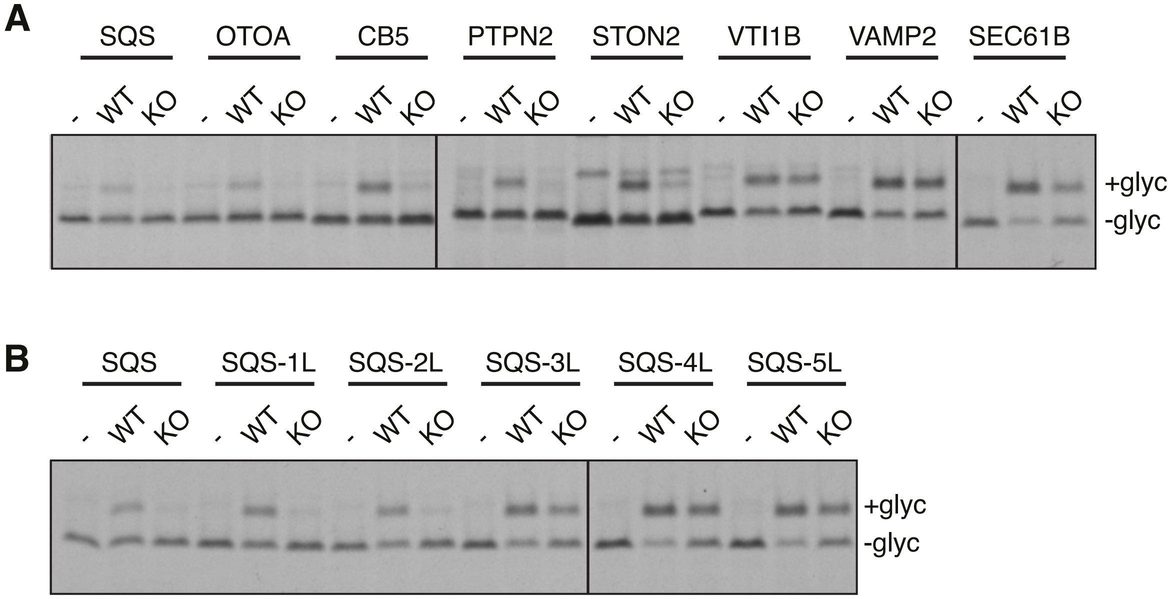

Fig. S16. Analysis of TA proteins for EMC-dependent insertion. (**A**) The indicated TA proteins were analyzed for insertion in hRMs from wild type or ΔEMC6 HEK293 cells. The proteins capable of interacting with TRC40 as seen in Fig. 1B were largely unaffected in the EMC6 knockout, while the others are almost entirely dependent on EMC6. Note that all of the proteins become ubiquitinated when they are not inserted in the membrane (*23, 24*). The mono-ubiquitinated product migrates slightly slower than the glycosylated product and is seen in some of the samples. This is most prominently observed in the case of STON2. (**B**) The indicated SQS mutants were analyzed for insertion in hRMs from wild type or ΔEMC6 HEK293 cells. As with the native proteins in panel A, the mutants capable of interacting with TRC40 as seen in Fig. 1E were unaffected by EMC6 knockout, while the others are almost entirely EMC-dependent.

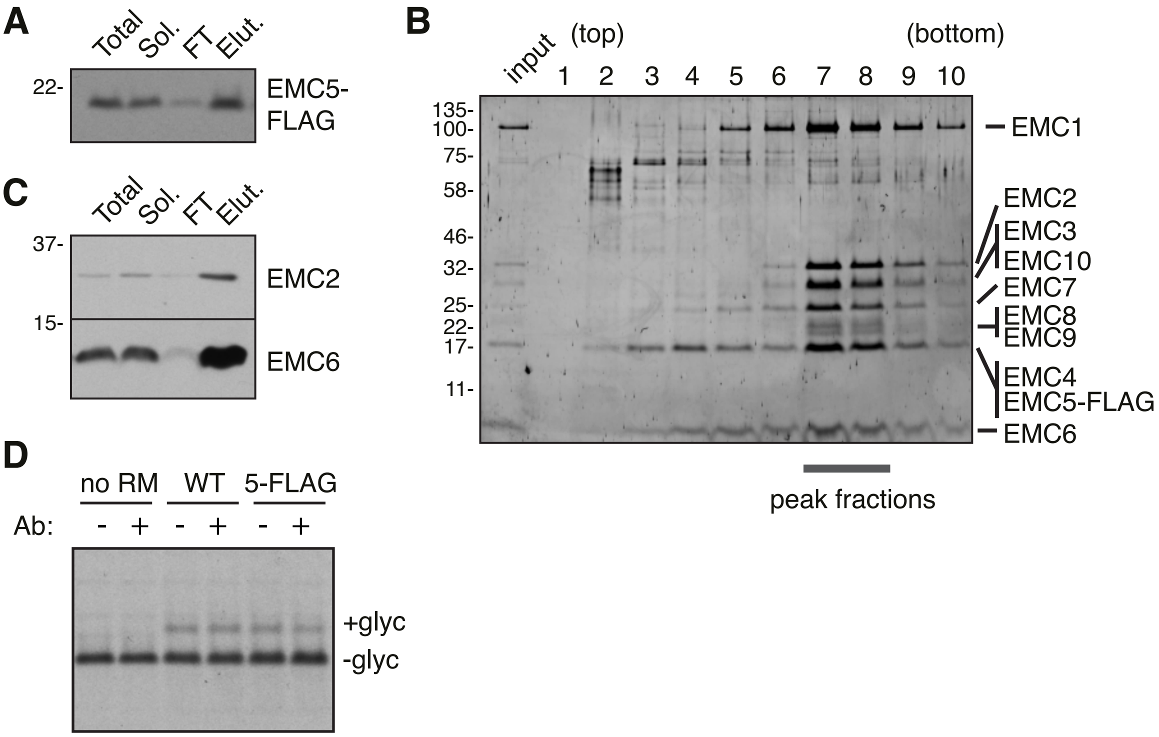

Fig. S17. Characterization of cells over-expressing EMC5-FLAG. (**A**) Cells stably over-expressing EMC5-FLAG for at least three generations were solubilized in digitonin and subjected to affinity purification using anti-FLAG resin. Aliquots of the purification at different steps are shown. Note that EMC is fully solubilized by digitonin ('sol' fraction), mostly depleted by the anti-FLAG resin (FT fraction), and recovered in the peptide elution. (**B**) A larger scale version of the purification in panel A was subjected to analysis by sucrose gradient. Note that all the subunits of the EMC (verified by mass spectrometry) co-sediment in the same fractions, and that very little free EMC5 is observed. Keratin contamination is seen in many of the lanes in the ~60-70 kD region. (**C**) Immunoblotting for EMC2 and EMC6 from a EMC5-FLAG purification as in panel A shows that the majority of EMC2 and EMC6 are depleted by removal of EMC5-FLAG. This suggests that the majority of EMC in these cells contains the over-expressed EMC5-FLAG, having displaced non-tagged endogenous EMC5. (**D**) Analysis of SQS insertion into microsomes from wild type HEK293 cells and EMC-FLAG over-expressing cells. The observation that EMC-FLAG cells are unimpaired in SQS insertion, despite nearly all EMC containing the FLAG tag, suggests that the FLAG-tagged complex is functional. The duplicate lanes for each condition represent samples without or with anti-FLAG antibody (Ab), which proved to have no effect on insertion.

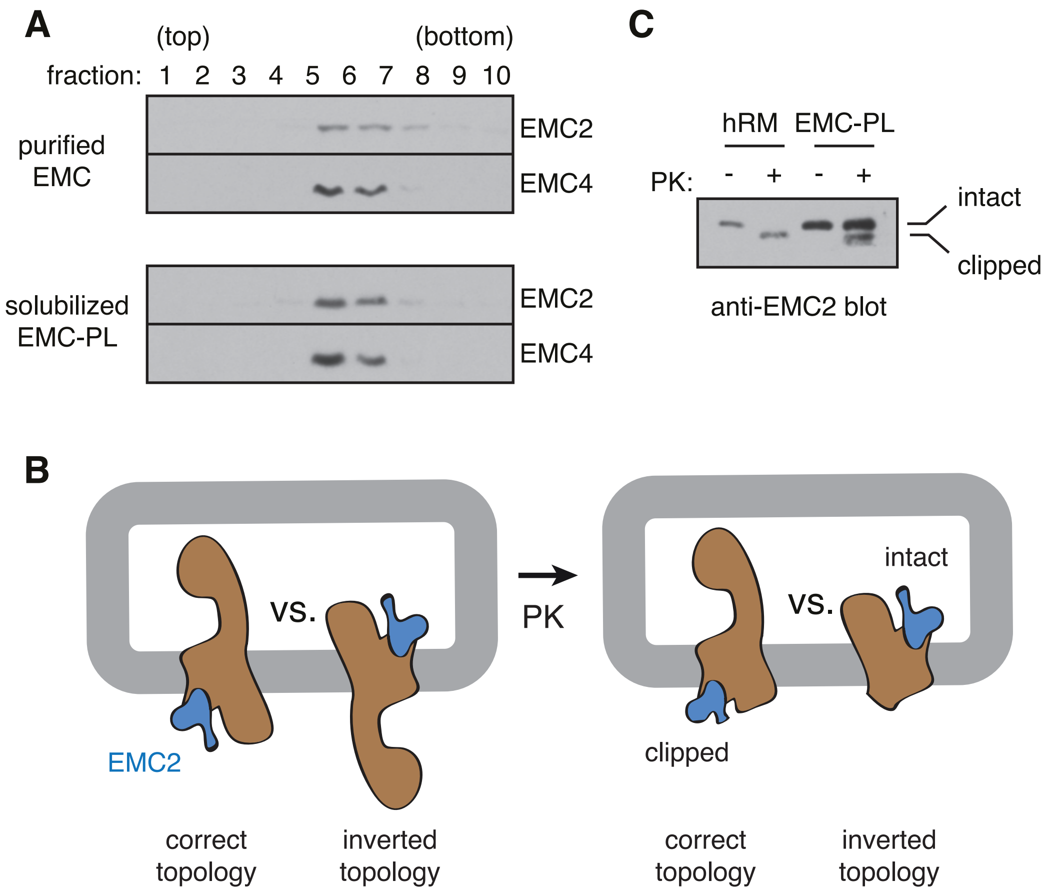

Fig. S18. Characterization of reconstituted EMC proteoliposomes. (**A**) Purified EMC in the detergent deoxyBigChap (DBC) was reconstituted in proteoliposomes for use in functional assays as in Fig. 4. An aliquot of the starting purified EMC and DBC-resolubilized proteoliposomes were separated on a sucrose gradient and the individual fractions immunoblotted for EMC2 and EMC4. Both subunits co-sediment in the same fractions before and after reconstitution, indicating that the complex in EMC-PL is intact. (**B**) Schematic of Proteinase K (PK) protection assay used to probe the topology of the reconstituted EMC. As shown in fig. S13A, EMC2 is ordinarily a peripheral membrane protein entirely on the cytosolic side of the ER. Thus, when EMC is in the correct topology, EMC2 is accessible to cytosolic PK, resulting in digestion to a stable core. By contrast, the inverted topology yields fully protected EMC2. Comparing levels of intact vs. clipped EMC2 after PK digest can then be used to indicate the proportion of EMC that has been incorporated in the correct orientation after reconstitution. **(C)** PK digests of intact hRMs compared to EMC-PL shows that all EMC is correctly oriented in hRM, but only around one-third is correctly oriented in the EMC-PL. This explains why between 2x and 4x EMC in proteoliposomes is needed to achieve levels of insertion comparable to native hRM (Fig. 4D).

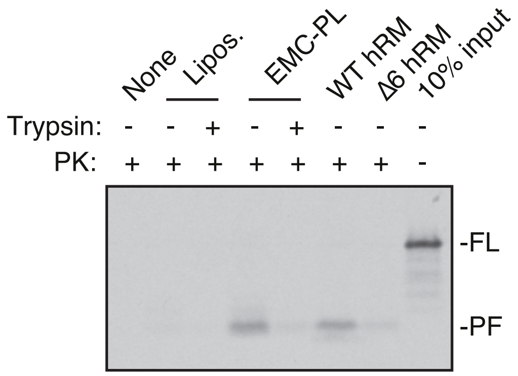

Fig. S19. SQS insertion depends on an intact EMC. Empty liposomes or EMC proteoliposomes (EMC-PL) were pre-teated with trypsin similarly to native hRMs (Fig 1F, fig S3D) to digest cytosolically exposed domains of the EMC. These vesicles, along with untreated controls, were tested for SQS insertion using the protease-protection assay (see Fig. 4B). The PURE system-derived SQS-CaM complex, an aliquot of which is shown in the last lane, was the substrate for these assays. Microsomes from wild type and ΔEMC6 HEK293 cells were used as controls. As in native microsomes (Fig. 1F), insertion of SQS into EMC-PL was trypsin sensitive, indicating that the process is protein mediated.

References and Notes

1. T. Kalbfleisch, A. Cambon, B. W. Wattenberg, A bioinformatics approach to identifying tail-anchored proteins in the human genome. *Traffic* **8**, 1687–1694 (2007). doi:10.1111/j.1600-0854.2007.00661.x Medline

2. R. S. Hegde, R. J. Keenan, Tail-anchored membrane protein insertion into the endoplasmic reticulum. *Nat. Rev. Mol. Cell Biol.* **12**, 787–798 (2011). doi:10.1038/nrm3226 Medline

3. A. Mateja, M. Paduch, H.-Y. Chang, A. Szydlowska, A. A. Kossiakoff, R. S. Hegde, R. J. Keenan, Structure of the Get3 targeting factor in complex with its membrane protein cargo. *Science* **347**, 1152–1155 (2015). doi:10.1126/science.1261671 Medline

4. M. Mariappan, A. Mateja, M. Dobosz, E. Bove, R. S. Hegde, R. J. Keenan, The mechanism of membrane-associated steps in tail-anchored protein insertion. *Nature* **477**, 61–66 (2011). doi:10.1038/nature10362 Medline

5. S. Stefer, S. Reitz, F. Wang, K. Wild, Y.-Y. Pang, D. Schwarz, J. Bomke, C. Hein, F. Löhr, F. Bernhard, V. Denic, V. Dötsch, I. Sinning, Structural basis for tail-anchored membrane protein biogenesis by the Get3-receptor complex. *Science* **333**, 758–762 (2011). doi:10.1126/science.1207125 Medline

6. F. Wang, E. C. Brown, G. Mak, J. Zhuang, V. Denic, A chaperone cascade sorts proteins for posttranslational membrane insertion into the endoplasmic reticulum. *Mol. Cell* **40**, 159–171 (2010). doi:10.1016/j.molcel.2010.08.038 Medline

7. M. Rao, V. Okreglak, U. S. Chio, H. Cho, P. Walter, S. O. Shan, Multiple selection filters ensure accurate tail-anchored membrane protein targeting. *eLife* **5**, e21301 (2016). doi:10.7554/eLife.21301 Medline

8. J. Rivera-Monroy, L. Musiol, K. Unthan-Fechner, Á. Farkas, A. Clancy, J. Coy-Vergara, U. Weill, S. Gockel, S.-Y. Lin, D. P. Corey, T. Kohl, P. Ströbel, M. Schuldiner, B. Schwappach, F. Vilardi, Mice lacking WRB reveal differential biogenesis requirements of tail-anchored proteins *in vivo*. *Sci. Rep.* **6**, 39464 (2016). doi:10.1038/srep39464 Medline

9. S. Brambillasca, M. Yabal, P. Soffientini, S. Stefanovic, M. Makarow, R. S. Hegde, N. Borgese, Transmembrane topogenesis of a tail-anchored protein is modulated by membrane lipid composition. *EMBO J.* **24**, 2533–2542 (2005). doi:10.1038/sj.emboj.7600730 Medline

10. B. M. Abell, M. Jung, J. D. Oliver, B. C. Knight, J. Tyedmers, R. Zimmermann, S. High, Tail-anchored and signal-anchored proteins utilize overlapping pathways during membrane insertion. *J. Biol. Chem.* **278**, 5669–5678 (2003). doi:10.1074/jbc.M209968200 Medline

11. M. Mariappan, X. Li, S. Stefanovic, A. Sharma, A. Mateja, R. J. Keenan, R. S. Hegde, A ribosome-associating factor chaperones tail-anchored membrane proteins. *Nature* **466**, 1120–1124 (2010). doi:10.1038/nature09296 Medline

12. S. Shao, M. C. Rodrigo-Brenni, M. H. Kivlen, R. S. Hegde, Mechanistic basis for a molecular triage reaction. *Science* **355**, 298–302 (2017). doi:10.1126/science.aah6130 Medline

13. S. Shao, R. S. Hegde, A calmodulin-dependent translocation pathway for small secretory proteins. *Cell* **147**, 1576–1588 (2011). doi:10.1016/j.cell.2011.11.048 Medline

14. N. Aviram, T. Ast, E. A. Costa, E. C. Arakel, S. G. Chuartzman, C. H. Jan, S. Haßdenteufel, J. Dudek, M. Jung, S. Schorr, R. Zimmermann, B. Schwappach, J. S. Weissman, M. Schuldiner, The SND proteins constitute an alternative targeting route to the endoplasmic reticulum. *Nature* **540**, 134–138 (2016). doi:10.1038/nature20169 Medline

15. S. Haßdenteufel, M. Sicking, S. Schorr, N. Aviram, C. Fecher-Trost, M. Schuldiner, M. Jung, R. Zimmermann, S. Lang, hSnd2 protein represents an alternative targeting factor to the endoplasmic reticulum in human cells. *FEBS Lett.* **591**, 3211–3224 (2017). Medline

16. J. G. Wideman, The ubiquitous and ancient ER membrane protein complex (EMC): Tether or not? *F1000Res.* **4**, 624 (2015). doi:10.12688/f1000research.6944.2 Medline

17. M. C. Jonikas, S. R. Collins, V. Denic, E. Oh, E. M. Quan, V. Schmid, J. Weibezahn, B. Schwappach, P. Walter, J. S. Weissman, M. Schuldiner, Comprehensive characterization of genes required for protein folding in the endoplasmic reticulum. *Science* **323**, 1693–1697 (2009). doi:10.1126/science.1167983 Medline

18. J. C. Christianson, J. A. Olzmann, T. A. Shaler, M. E. Sowa, E. J. Bennett, C. M. Richter, R. E. Tyler, E. J. Greenblatt, J. W. Harper, R. R. Kopito, Defining human ERAD networks through an integrative mapping strategy. *Nat. Cell Biol.* **14**, 93–105 (2011). doi:10.1038/ncb2383 Medline

19. M. Richard, T. Boulin, V. J. P. Robert, J. E. Richmond, J.-L. Bessereau, Biosynthesis of ionotropic acetylcholine receptors requires the evolutionarily conserved ER membrane complex. *Proc. Natl. Acad. Sci. U.S.A.* **110**, E1055–E1063 (2013). doi:10.1073/pnas.1216154110 Medline

20. T. Satoh, A. Ohba, Z. Liu, T. Inagaki, A. K. Satoh, dPob/EMC is essential for biosynthesis of rhodopsin and other multi-pass membrane proteins in *Drosophila* photoreceptors. *eLife* **4**, (2015). doi:10.7554/eLife.06306 Medline

21. R. J. Louie, J. Guo, J. W. Rodgers, R. White, N. Shah, S. Pagant, P. Kim, M. Livstone, K. Dolinski, B. A. McKinney, J. Hong, E. J. Sorscher, J. Bryan, E. A. Miller, J. L. Hartman IV, A yeast phenomic model for the gene interaction network modulating CFTR-ΔF508 protein biogenesis. *Genome Med.* **4**, 103 (2012). doi:10.1186/gm404 Medline

22. S. Lahiri, J. T. Chao, S. Tavassoli, A. K. O. Wong, V. Choudhary, B. P. Young, C. J. R. Loewen, W. A. Prinz, A conserved endoplasmic reticulum membrane protein complex (EMC) facilitates phospholipid transfer from the ER to mitochondria. *PLOS Biol.* **12**, e1001969 (2014). doi:10.1371/journal.pbio.1001969 Medline

23. T. Hessa, A. Sharma, M. Mariappan, H. D. Eshleman, E. Gutierrez, R. S. Hegde, Protein targeting and degradation are coupled for elimination of mislocalized proteins. *Nature* **475**, 394–397 (2011). doi:10.1038/nature10181 Medline

24. E. Itakura, E. Zavodszky, S. Shao, M. L. Wohlever, R. J. Keenan, R. S. Hegde, Ubiquilins chaperone and triage mitochondrial membrane proteins for degradation. *Mol. Cell* **63**, 21–33 (2016). doi:10.1016/j.molcel.2016.05.020 Medline

25. A. S. Anghel, P. T. McGilvray, R. S. Hegde, R. J. Keenan, *Cell Reports*, in press.

26. F. Wang, C. Chan, N. R. Weir, V. Denic, The Get1/2 transmembrane complex is an endoplasmic-reticulum membrane protein insertase. *Nature* **512**, 441–444 (2014). doi:10.1038/nature13471 Medline

27. G. Savidis, W. M. McDougall, P. Meraner, J. M. Perreira, J. M. Portmann, G. Trincucci, S. P. John, A. M. Aker, N. Renzette, D. R. Robbins, Z. Guo, S. Green, T. F. Kowalik, A. L. Brass, Identification of Zika virus and dengue virus dependency factors using functional genomics. *Cell Reports* **16**, 232–246 (2016). doi:10.1016/j.celrep.2016.06.028 Medline

28. Y. Shimizu, T. Ueda, PURE technology. *Methods Mol. Biol.* **607**, 11–21 (2010). doi:10.1007/978-1-60327-331-2_2 Medline

29. S. Stefanovic, R. S. Hegde, Identification of a targeting factor for posttranslational membrane protein insertion into the ER. *Cell* **128**, 1147–1159 (2007). doi:10.1016/j.cell.2007.01.036 Medline

30. J. L. Garrison, E. J. Kunkel, R. S. Hegde, J. Taunton, A substrate-specific inhibitor of protein translocation into the endoplasmic reticulum. *Nature* **436**, 285–289 (2005). doi:10.1038/nature03821 Medline

31. O. Chakrabarti, R. S. Hegde, Functional depletion of mahogunin by cytosolically exposed prion protein contributes to neurodegeneration. *Cell* **137**, 1136–1147 (2009). doi:10.1016/j.cell.2009.03.042 Medline

32. A. L. MacKinnon, J. L. Garrison, R. S. Hegde, J. Taunton, Photo-leucine incorporation reveals the target of a cyclodepsipeptide inhibitor of cotranslational translocation. *J. Am. Chem. Soc.* **129**, 14560–14561 (2007). doi:10.1021/ja076250y Medline

33. A. Krogh, B. Larsson, G. von Heijne, E. L. Sonnhammer, Predicting transmembrane protein topology with a hidden Markov model: Application to complete genomes. *J. Mol. Biol.* **305**, 567–580 (2001). doi:10.1006/jmbi.2000.4315 Medline

34. G. Zhao, E. London, An amino acid “transmembrane tendency” scale that approaches the theoretical limit to accuracy for prediction of transmembrane helices: Relationship to biological hydrophobicity. *Protein Sci.* **15**, 1987–2001 (2006). doi:10.1110/ps.062286306 Medline

35. G. Zhao, E. London, Strong correlation between statistical transmembrane tendency and experimental hydrophobicity scales for identification of transmembrane helices. *J. Membr. Biol.* **229**, 165–168 (2009). doi:10.1007/s00232-009-9178-0 Medline

36. A. Sharma, M. Mariappan, S. Appathurai, R. S. Hegde, In vitro dissection of protein translocation into the mammalian endoplasmic reticulum. *Methods Mol. Biol.* **619**, 339–363 (2010). doi:10.1007/978-1-60327-412-8_20 Medline

37. P. Walter, G. Blobel, Preparation of microsomal membranes for cotranslational protein translocation. *Methods Enzymol.* **96**, 84–93 (1983). doi:10.1016/S0076-6879(83)96010-X Medline

38. Z.-R. Zhang, J. S. Bonifacino, R. S. Hegde, Deubiquitinases sharpen substrate discrimination during membrane protein degradation from the ER. *Cell* **154**, 609–622 (2013). doi:10.1016/j.cell.2013.06.038 Medline

39. H. R. Pelham, R. J. Jackson, An efficient mRNA-dependent translation system from

reticulocyte lysates. *Eur. J. Biochem.* **67**, 247–256 (1976). doi:10.1111/j.1432-1033.1976.tb10656.x Medline

40. T. J. Schoenmakers, G. J. Visser, G. Flik, A. P. Theuvenet, CHELATOR: An improved method for computing metal ion concentrations in physiological solutions. *Biotechniques* **12**, 870–874, 876–879 (1992). Medline

41. J. W. Chin, A. B. Martin, D. S. King, L. Wang, P. G. Schultz, Addition of a photocrosslinking amino acid to the genetic code of *Escherichia coli*. *Proc. Natl. Acad. Sci. U.S.A.* **99**, 11020–11024 (2002). doi:10.1073/pnas.172226299 Medline

42. T. Kobayashi, O. Nureki, R. Ishitani, A. Yaremchuk, M. Tukalo, S. Cusack, K. Sakamoto, S. Yokoyama, Structural basis for orthogonal tRNA specificities of tyrosyl-tRNA synthetases for genetic code expansion. *Nat. Struct. Biol.* **10**, 425–432 (2003). doi:10.1038/nsb934 Medline

43. F. A. Ran, P. D. Hsu, J. Wright, V. Agarwala, D. A. Scott, F. Zhang, Genome engineering using the CRISPR-Cas9 system. *Nat. Protoc.* **8**, 2281–2308 (2013). doi:10.1038/nprot.2013.143 Medline

44. P. de Felipe, G. A. Luke, L. E. Hughes, D. Gani, C. Halpin, M. D. Ryan, *E unum pluribus*: Multiple proteins from a self-processing polyprotein. *Trends Biotechnol.* **24**, 68–75 (2006). doi:10.1016/j.tibtech.2005.12.006 Medline
